# FLPSO-AMPS: an optimized WSN model for air quality monitoring in tier-2 smart cities

**DOI:** 10.1038/s41598-025-19748-3

**Published:** 2025-10-15

**Authors:** Lingaraj K., Rashmi Laxmikant Malghan, Karthik Rao M. C., Lalit Garg, Somanath Swamy R H M, Vishwanatha H. M.

**Affiliations:** 1 Department of Computer Science and Engineering, Rao Bahadur Y Mahabaleswarappa Engineering College, Ballari, 583104 Karnataka India; 2 Manipal Institute of Technology, Manipal Academy of Higher Education, Manipal, 576104 Karnataka India; 3 Department of Mechatronics, Manipal Institute of Technology, Manipal Academy of Higher Education, Manipal, 576104 Karnataka India; 4 Department of Computer Information System, University of Malta, Msida, 2080 Malta; 5 Department of Mechanical Engineering, Rao Bahadur Y Mahabaleswarappa Engineering College, Ballari, 583104 Karnataka India; 6 Department of Mechanical and Industrial Engineering, Manipal Institute of Technology, Manipal Academy of Higher Education, Manipal, 576104 Karnataka India; 7 Faculty of Information Technology, Industrial University of Ho Chi Minh, Ho Chi Minh City, Vietnam

**Keywords:** Energy science and technology, Engineering, Environmental sciences, Mathematics and computing

## Abstract

Wireless Sensor Networks (WSNs) are composed of small, cost-effective sensing nodes that are primarily employed for the collection of environmental data. These networks are integral to various applications including industrial pollution monitoring, disaster management, and air quality regulation. However, WSNs encounter significant challenges, such as energy efficiency, end-to-end delay, and packet loss during data transmission. Existing methodologies often fall short in optimizing the network lifespan while ensuring reliable data delivery. To address these limitations, this study introduces FLPSO-AMPS, a novel Fuzzy Logic-based Particle Swarm Optimization (FLPSO) approach aimed at enhancing energy-efficient routing in WSN-based Air Pollution Monitoring Systems (APMS) for Tier-2 smart cities. The proposed approach leverages fuzzy logic principles combined with PSO to intelligently select optimal routing paths, thereby ensuring minimal energy consumption and enhanced network longevity. Unlike conventional methodologies, FLPSO-AMPS incorporates real-time pollutant data collection and mobility-aware optimization to improve network performance. The effectiveness of FLPSO-AMPS was validated through extensive simulations, demonstrating superior performance over existing approaches, particularly with improvements of 10% in energy efficiency, 15% in task delay, 24.5% in packet delivery ratio (PDR), 11.5% in packet loss ratio (PLR), and 20.1% in throughput. These findings underscore the potential of FLPSO-AMPS in establishing an intelligent, resource-efficient air quality monitoring framework for smart cities. Future research will explore security enhancements to safeguard data transmissions in APMS networks.

## Introduction

The use of Wireless Sensor Networks (WSNs) is gaining global popularity. There is an immense need for significant improvement in flood disaster monitoring^1^. The use of WSN for disaster monitoring presents certain benefits including cost-effectiveness, ease of management, and the ability to expand networks^2^. Furthermore, the capricious characteristics of natural calamities impede the dissemination of pertinent data to related sensor nodes, with minimal latency. A WSN is composed of several wirelessly linked sensor nodes. The proper functioning of software and hardware is contingent on their ability to adapt to new circumstances and meet evolving demands. These sensors exhibit diminutive size and restricted storage and processing capabilities. Owing to limited transmission energy, WSNs can communicate within a restricted range with specific neighboring nodes. According to^3^, nodes are dependent on cooperation to carry out various tasks, such as sensing, signal processing, routing, localization, and security^4^.

An air pollution monitoring system (APMS) is a crucial factor that impacts the standards of living, human well-being, and the ecosystem^5^. Typically, oversight of an air pollution monitoring program falls under the purview of the pollution control department and entails significant expenses. The use of wireless methodology is deemed a viable option for air pollution monitoring schemes owing to progress in communication technology. The implementation of an APMS involves the utilization of technologies, such as GSM and GPRS, which are associated with significant expenses in terms of both maintenance and installation. The implementation of an APMS offers benefits for monitoring various high-risk regions within a country^6^. This is achieved through the provision of real-time data and alerts in the event of significant air quality degradation. Organizations have the potential to utilize these data to swiftly implement measures such as, evacuating specific areas or dispatching emergency response teams. The implementation of a wireless APMS involves the use of an air quality index(AQI) to categorize the distinct air pollution locations. The advancement of technology, particularly WSN, has transformed air pollution monitoring (APM) into a sophisticated smart air pollution monitoring system (SAPMS). This has facilitated monitoring of environmental parameters with greater precision and efficacy, thereby mitigating pollution and other undesirable environmental effects^7,8^.

To address these challenges, this study introduces FLPSO-AMPS, a fuzzy logic-based particle swarm optimization (FLPSO) approach aimed at enhancing energy-efficient routing in air pollution monitoring systems (APMS) for tier-2 smart cities. Traditional routing methods for WSN often exhibit limited adaptability to dynamic network conditions, resulting in suboptimal energy utilization and compromised data reliability^9,10^. The proposed FLPSO-AMPS algorithm employs fuzzy logic principles and particle swarm optimization (PSO) to intelligently determine optimal network routes, thereby ensuring reduced energy consumption, enhanced data transmission reliability, and a prolonged network lifespan^11,12^.This study provides an area for combining WSN technology with advanced efficiency approaches, effectively addressing the limitations of existing air pollution monitoring systems. The key contributions of this study include the development of an optimized routing scheme that improves data transmission efficiency in a WSN-based APMS. The implementation of a real-time air pollution monitoring system utilizing WSN nodes to track pollutants, such as AQI, PM2.5, PM10, CO, $SO_2$, and $NO_2$, and a comparative evaluation of the proposed method against existing state-of-the-art approaches in terms of PDR, EDP, PLR, and throughput^13,14^. By providing a scalable and resource-efficient framework, FLPSO-AMPS offers a cost-effective solution for real-time pollution detection and monitoring, making a valuable contribution to smart city development. This study underscores the necessity for intelligent and adaptive routing strategies in WSN-based APMS and presents a novel approach that enhances network efficiency while ensuring reliable environmental monitoring. The proposed method bridges the gap between traditional energy-intensive routing protocols and modern optimization techniques, enabling smart cities to deploy sustainable and high-performance air quality monitoring systems^15,16^. The findings of this study have the potential to improve urban air-quality monitoring, thereby contributing to environmental sustainability and public health protection.

## Related work

###  Air pollution monitoring system

This section outlines various techniques utilized for ineffective routing within the WSN and highlights the constraints of the study. Divya et al.^17^ - designed a technique for monitoring AQI in WSN environments, highlighting the need for further research in this critical field. The proposed study advocates the development of a smart city that focuses on mitigating pollution and addressing environmental issues. Maurya et al.^18^, implemented an APMS that was both cost-effective and reliable for use in developing countries. The scheme for monitoring pollutants in the atmosphere quantified the concentrations of CO and other toxic gases. The methodology relies on the use of the GPS and PRS. Furthermore, the analog information collected by the sensor was transmitted to the microcontroller unit, where it was converted into digital information and sent to a remote server in the cloud via a GPRS scheme, along with the corresponding positional information. The proposed methodology exhibited enhanced reliability owing to its reliance on the global positioning system. Siregar et al.^19^ conducted air pollution monitoring and analysis at a specific location. The results were communicated to the administrator through a graphical user interface (GUI) utilizing an internet network. The protocol employed air pollution investigation approach based on the index standard panacea urara (ISPU), which has been previously utilized in indonesia. The research site has undergone an analysis of the concentrations of several gases, including CO2 and NO2, according to the results of the conducted tests. Handayani et al.^20^ focused on the automation of air monitoring in enclosed parking areas by evaluating the exhaust emissions from motor vehicles. The present research focused on the development of a real-time operating system that is precise, resource-efficient, portable, reliable, cost-effective and compatible with interconnected networks that rely on internet protocols. The study presented herein offers pertinent information to the general populace, particularly individuals who frequently live in densely populated areas, with the aim of mitigating atmospheric contamination.

Agnihotri et al.^21^ extensively reviewed various studies and presented comprehensive insights into the components and constituents involved in the development of WSN. Wireless sensor network monitoring strategies have the potential to be expanded to encompass additional forms of pollution detection, such as those related to water, soil, and radioactive contamination. Moreover, the utilization of a communication protocol with high efficiency can facilitate the acquisition of information in a real-time setting, whereas the acquisition of information processing may be explored in future endeavors. Marques et al.^22^ conducted a study on a stand-alone robot equipped with an air boot that demonstrated the ability to monitor indoor air quality. The study focused on corporations’ robotic systems that possess application programming interfaces to facilitate the dissemination of data on social media platforms and enable inter-connectivity with indoor monitoring systems. Guanochanga et al.^23^ outlined the initial steps taken to implement a WSN to monitor air quality metrics in intelligent urban services. The main aim of this research was to design a wireless framework with minimal cost. IoT facilitates the visualization of a highly interconnected network of web applications with a GUI at an advanced level. The researchers investigated the feasibility of utilizing tangible instruments for gathering APM data in real-time and generating behavioral models of air pollution scenarios in urban areas. Naik et al.^24^ proposed the implementation of smart cities, particularly in terms of environmental monitoring, vehicular maintenance, and traffic management^25^. Hojaiji et al.^26^ presented a framework for APMS schemes that enabled real-time data collaboration.

Laskar et al.^27^ developed a surveillance system that focuse on air pollution. The system utilities an optimization technique to identify the global minimum based on real-time pollution data obtained from a wireless sensor network. A supplementary tool was activated to delineate pollution boundaries and suggest alternative pathways within a vast urban network, thereby assisting operators in determining the most efficient route to mitigate the spread of illness. Adumanu^28^ utilized various WSN to assess real-time water quality, resulting in an optimized data communication schedule that conserves energy. In additionally, solar panels were used to improve the duration of the sensor hop. Gazis et al.^29^ proposed the use of WSN to monitor underground routes that are covered and box-shaped to protect wildlife. The fundamental goal of this research was to implement commonly positioned routes and techniques and employ information synthesis to develop inexpensive and efficient tiny sensors that align with typical animal movement patterns. Luo et al.^30^ performed studies on sensors and pollution monitoring network schemes, as well as different techniques for pollution recognition. Subsequently, a thorough examination of the models pertaining to the identification of contaminated resources was provided and interconnected. This study expounded upon certain constraints associated with the utilization of sensor systems in the context of the APM. The aforementioned survey revealed that the monitoring process incurred significant costs, owing to notable spatial and temporal inconsistencies. They employed a hybrid approach to schedule and deploy distinct features of the air pollution approach. Subsequently, a real-time air quality model was generated based on sensor readings. In addition, optimization techniques are utilized to assess the physical characteristics of the dispersion of air pollutants and to incorporate factual data obtained from contemporary pollution detection systems. Pavani et al.^31^ devised a practical pollution monitoring system that utilizes a WSN to track the concentration levels of CO in a real-time setting. The experimental setup consisted of a testbed while five sequential hops, wherein CO sensors were calibrated to account for variations in the CO concentration levels^32,33^. Furthermore, an alert system dispatches emails and messages to inform the system administrator of increasing CO levels at the earliest opportunity s^34–36^.

Recent works further enrich this landscape. Singh et al.^37^ introduced a hybrid genetic algorithm with greedy mutation for heterogeneous IoT-enabled WSNs, achieving over 30% improvement in lifetime compared to GA-based baselines . Complementarily, Del-Valle-Soto et al.^38^ provided a systematic survey of clustering routing protocols using metaheuristic techniques, categorizing original, modified, and hybrid strategies, and highlighting bio-inspired methods as promising directions for energy-efficient routing. In the development of an optimized Wireless Sensor Network (WSN) model for air quality monitoring in tier-2 smart cities, several advanced technologies and frameworks play a crucial role^39–41^ . One way to solve these environmental monitoring issues is by using IoT sensors, data processing algorithms and communication infrastructures^42^. Advanced communication technologies like 5g is that it enabled the use of IoT devices with real-time monitoring. This new implementation would help in live data collection and analysis to solve urban problems like network overload and security^43^. Furthermore, the IoT framework uses artificial intelligence and data analytics for efficient data management and rapid response to environmental changes^44^. It is imperative to also address data quality issues when monitoring air quality. To enhance the quality of time series data, a two-phase framework was proposed to detect outliers and to deal with the missing value utilizing machine learning algorithms and interpolation techniques^45^. The methodologies here ensure that air quality data is consistent and reliable, which is important for smart city applications. AI is not limited to just processing data; they are used in generative spatial AI models for urban planning and analysis. This makes predicting much easier and taking decisions easier and it also makes urban environment sustainable and resilient^46^. At the same time, another important feature to have in place are privacy-preserving authentication schemes so as to secure the data which is gleaned from IoT sensors. It is important so as to prevent unauthorized access while ensuring that data integrity is maintained^47^. In smart cities tier-2, scalable and sustainable solutions with the combination of smart grids and renewable energy solutions are also essential. This integration solves sustainability, security, and affordability trilemma and enhances the efficiency of using resources^48^.

The WSNs were used to illustrate the effectiveness of multi mobile agent (MMA) based on data collection and aggregation. The installation of these agents must be carefully planned and tailored to the unique characteristics of WSN in order to guarantee their effectiveness^49–51^. Prior studies primarily focused on ascertaining the number of mobile agent (MA) to be implemented and the configuration of SNs for each MA^52^. This study involved tracing the origin nodes of each MA to map their respective journeys. These algorithms were employed to evaluate the effectiveness of the aggregation. The present research introduces a methodology for consolidating network nodes to determine the optimal route, considering multiple agents, energy conservation, and aggregation effectiveness within a minimal time-frame. This study also highlights the constraints of these techniques when applied to extensive networks.

The predominant focus of contemporary research in the domain of WSN pertains to the minimization of energy consumption in the course of route design. The migration approach of an MA can have a substantial impact on energy usage and network durability, as indicated by previous studies^53–55^. Determining the optimal configuration of SNs for traversal using MAs can present a formidable task.The purpose of this investigation was to find a solution to the challenges associated with IP in networks by applying MMA methodology. The innovative methodology for WSN consider the requirements of a satisfactory quantity of MA, an effective clustering of nodes, and ultimately the criterion of optimal route selection for each MA to cover all of its assigned SNs.

Therefore, a centralized planning model was suggested, wherein the sink node determines the entire route schedule instead of being limited by energy consumption, as observed in previous systems. The current methodology presents certain concerns pertaining to data aggregation, specifically regarding the consolidation of all sensor nodes into a singular cluster, which warrants careful consideration. The selection of an appropriate sensor node to serve as a cluster head is a crucial consideration in the context of data aggregation, as it has implications for the extension of the network’s operational duration. Optimal itinerary planning pertaining to the extension of the sensor network lifespan and the concomitant reduction of data collection time warrants significant attention. The CM-FFA algorithm is suggested as a means of selecting an appropriate sensor node to serve as the cluster head. The BM-FPA algorithm^56^ fails to address hot-spot issues, as per experimental studies.Table 1 demonstrates different air polloution monitoring systems

**Table 1:** Comparison of different air polloution monitoring system.

**Refs**	**Approach**	**Advantages**	**Research gap**
Handayani et al.^20^	Monitoring in enclosed parking areas	Real-time, cost-effective, and portable	Hardware design and power-related attributes
Agnihotri et al.^21^	Review on WSN in pollution detection	Comprehensive insights on WSN components	Expansion to other forms of pollution detection
Marques et al.^22^	Robot equipped with air boot	Indoor air quality, API for data sharing	High computational complexity
Guanochanga et al.^23^	WSN for air quality in intelligent urban services	Cost-effective, real-time data	Potential for broader application
Naik et al.^24^	Gas-sensing components	Implementation in smart cities, multiple applications	Potential for broader application
Dixit al.^32^	E2R2D2: Energy-Efficient Robust Routing and Data Distribution Protocol for WSN-based Air Pollution Monitoring	Enhanced energy efficiency and robust data routing in dynamic WSN environments	Lack of integration with mobile agents and fuzzy optimization for real-time adaptability
Abdul kareem et al.^33^	WSN-based air pollution monitoring system for university workstation	Cost-effective monitoring for small-scale and localized environments	Limited scalability, lacks advanced routing and optimization mechanisms
Shi et al.^35^	Mechanism model combined with deep learning for indoor air pollution prediction	Accurate prediction using hybrid models in residential/commercial spaces	Indoor-focused; no real-time outdoor deployment or routing optimization
Berniak-Woźny et al.^39^	Start-up–driven sustainability framework for incubator–city collaboration in smart cities	Encourages innovation ecosystems and stakeholder partnerships for sustainable growth	Lacks integration with IoT/AI-driven environmental monitoring and technical deployment
Madan et al.^40^	IoT with Elman neural network for smart waste management & pollution forecasting	Enables predictive waste/air-quality analysis with neural forecasting	Limited scalability tests; energy efficiency and sensor reliability not addressed
Sahu et al.^41^	Multi-objective optimization (digital twin + MDP + PSO) for smart parking	Optimizes parking with Pareto fronts, enhancing urban mobility	Focused on parking; does not extend to environmental sensing or WSN scalability
Huo et al.^42^	AI-based quasi-natural experiment for green resilience in smart cities	Shows AI benefits in sustainability and adaptive resilience	Primarily policy-level; lacks detailed IoT/WSN-level deployment
Mahomed & Saha^43^	Real-time digital twin integration with 5G	Achieves real-time responsiveness and reduced latency in smart city monitoring	Security, energy cost, and interoperability with low-cost WSN nodes remain gaps
Salih et al.^44^	IoT in urban development: applications, case studies, challenges	Provides a comprehensive landscape of IoT-enabled smart city implementations	Broad overview; limited quantitative validation for air-quality monitoring
Alsalehy & Bailey^45^	Two-phase ML framework for outlier detection and missing value imputation	Improves reliability of time-series gas/weather data	Focused on data preprocessing; no routing/energy-efficiency integration for WSNs
Huang et al.^46^	Generative spatial AI models for urban digital twin	Facilitates predictive urban planning with spatial AI	Limited to planning; lacks integration with sensor reliability or real-time routing
Kim et al.^47^	Privacy-preserving authentication using PUF + biometrics	Ensures data integrity and prevents unauthorized IoT access	Overheads on constrained WSN nodes not fully studied; deployment feasibility unclear
Silva et al.^48^	Literature review on smart grids within smart cities	Identifies sustainability and resource efficiency gains through grid–IoT integration	Mainly grid-focused; limited discussion on environmental sensing and low-cost sensor networks

###  Differences between the existing and proposed work

Existing air pollution monitoring systems within Wireless Sensor Networks (WSNs) predominantly utilize conventional routing techniques, which are frequently constrained by high energy consumption, inefficient data transmission, and limited scalability. These methodologies often lack intelligent decision-making mechanisms, resulting in suboptimal network performance and diminished sensor longevity. Furthermore, many existing methods do not adequately address the real-time dynamic nature of environmental monitoring, thereby reducing their efficacy in urban environments where pollutant levels fluctuate rapidly due to industrial and vehicular emissions. In contrast to traditional methodologies, the FLPSO-AMPS approach to enhance routing and data transmission in WSN-based APMS. The primary distinctions between existing techniques and the proposed approach are as follows:

1. Incorporation of Advanced Pollutant Detection : Unlike previous approaches that primarily monitor a limited set of pollutants such as CO, $NO_2$ and $SO_2$, FLPSO-AMPS encompasses a broader spectrum of environmental contaminants, including ethane, acetylene, methanol, formaldehyde, formic acid, ammonia, naphthalene, and lead, thereby ensuring a more comprehensive assessment of air quality.
2. Intelligent and Adaptive Routing Mechanism : Existing systems typically employ fixed or heuristic-based routing algorithms that do not dynamically adapt to network conditions. In contrast, FLPSO-AMPS utilizes fuzzy logic and PSO, facilitating real-time adaptation to changes in network topology, optimizing energy consumption, and enhancing data transmission reliability.
3. Enhanced Scalability and Real-Time Performance: Traditional methods encounter challenges in real-time data collection due to limited computing resources and high communication overhead. The FLPSO-AMPS framework optimizes the energy efficiency of multi-mobile agent routing, enabling scalable deployment in Tier-2 smart cities, where cost-effective and efficient solutions are imperative.
4. Comparison with SOTA Techniques : The proposed method is rigorously evaluated against well-established PSO-based routing techniques such as ELDC and IEESEP. Simulation results demonstrate that FLPSO-AMPS consistently outperforms these methods, achieving 10% better energy efficiency, a 15% reduction in task delay, a 24.5% higher PDR, an 11.5% lower PLR, and a 20.1% increase in throughput.

By integrating fuzzy logic with PSO-based optimization, the FLPSO-AMPS framework offers a more energy-efficient, adaptive, and scalable solution for real-time APM. The suggested technology is designed to support smart city applications, enabling policymakers and environmental agencies to make data-driven decisions for pollution control and urban planning.

## Fuzzy logic-based particle swarm optimization in air quality monitoring system

The main objective of this research is to investigate the effects of air pollution on human health. Numerous health disorders that cause a wide range of illnesses, have been associated with air pollution. Air pollution is a crucial determinant of both human health and environmental concerns and encompasses a diverse array of hazardous gases, chemicals, and minute particulate matter. The primary sources of these pollutants are vehicle and industrial emissions as well as volatile organic compounds. Air pollution is a significant peril to human health; therefore, it is imperative to eradicate air pollution as a fundamental requirement for achieving optimal health. Researchers conducted an investigation utilizing mobile sensors to explore the feasibility of implementing a mobile sensor network for environmental monitoring in smart cities. Additionally, they examined the impact of mobility on the measurement accuracy. Diverse data sources, encompassing both objective (derived from sensors) and subjective (reported by PCD), are integrated to generate significant indicators and facilitate discourse between the wider populace and municipal authorities.

AQI indicators are derived exclusively from objective data. The sensor array gathered various environmental indicators, including but not limited to $CO_2$, hydrocarbons, $O_3$, and PM pollutants. The metrics, which rely exclusively on empirical data, are subsequently disseminated to diverse stakeholders, such as the general population, researchers, and local government officials. The level of air quality (LAQ) is used to determine the concentration of pollutants in the air :

1 $$\begin{aligned} LAQ = { \bigg (\frac{L_{max}- L_{min}}{P_{max}- P_{min}} \bigg ) } \times ({P_{max}- P_{min}}) + L_{min} \end{aligned}$$

As demonstrated by Eq.(1), there exists a discernible disparity between the maximum and minimum LAQ values, denoted as $L_{max}$ and $L_{min}$, respectively. The sensor denoted as $P_{min}$ exhibited the lowest level of pollution, while the sensor designated as $P_{max}$ demonstrated the highest degree of pollution. The results indicate that the sensor with the least amount of pollution exhibits the lowest LAQ values, whereas the sensor with the highest level of pollution displays the highest LAQ values, as demonstrated in Eq. (1) The acquisition of reliable information regarding pollution in a specific area can be achieved using sensors and the subsequent indicators they offer.

The air quality of a specific region m was determined as follows:

2 $$\begin{aligned} RE=\sqrt{ \frac{\sum _{k=1}^{|m|} {{(\overline{z} + z )}^{2}} }{m} } \end{aligned}$$

The accuracy of model $\overline{z}$ compared to the actual truth of model z, as given in Eq. (2), in accordance with the RE.

Surveillance of the surroundings in which collections are stored is of utmost importance in order to obtain an understanding of and assess the conditions under which they are maintained. The use of a time-series model enables the simulation of the prospective behavior of a system, thereby providing short-term predictions denoted as ${\Delta }_r(\Theta )$ and prediction ranges p and E for the stressors in question.

3 $$\begin{aligned} {\Delta }_r(\Theta ) = { P(x; \Theta ) { y_r} + E(x; \Theta ) { z_r} } \end{aligned}$$

Eq. (3) demonstrates that (x; $\Theta$) denotes the estimate error, ${ y_r}$ is the nonlinear regression, and ${ z_r}$ denotes the parameter vector. Pollution has an impact on land, air, and water on earth by releasing contaminants into these three environments. Some types of pollutant released into the atmosphere include smog, contaminated air, and toxic gas emissions. In addition to natural sources, human activities can lead to pollution.

**Fig. 1:**
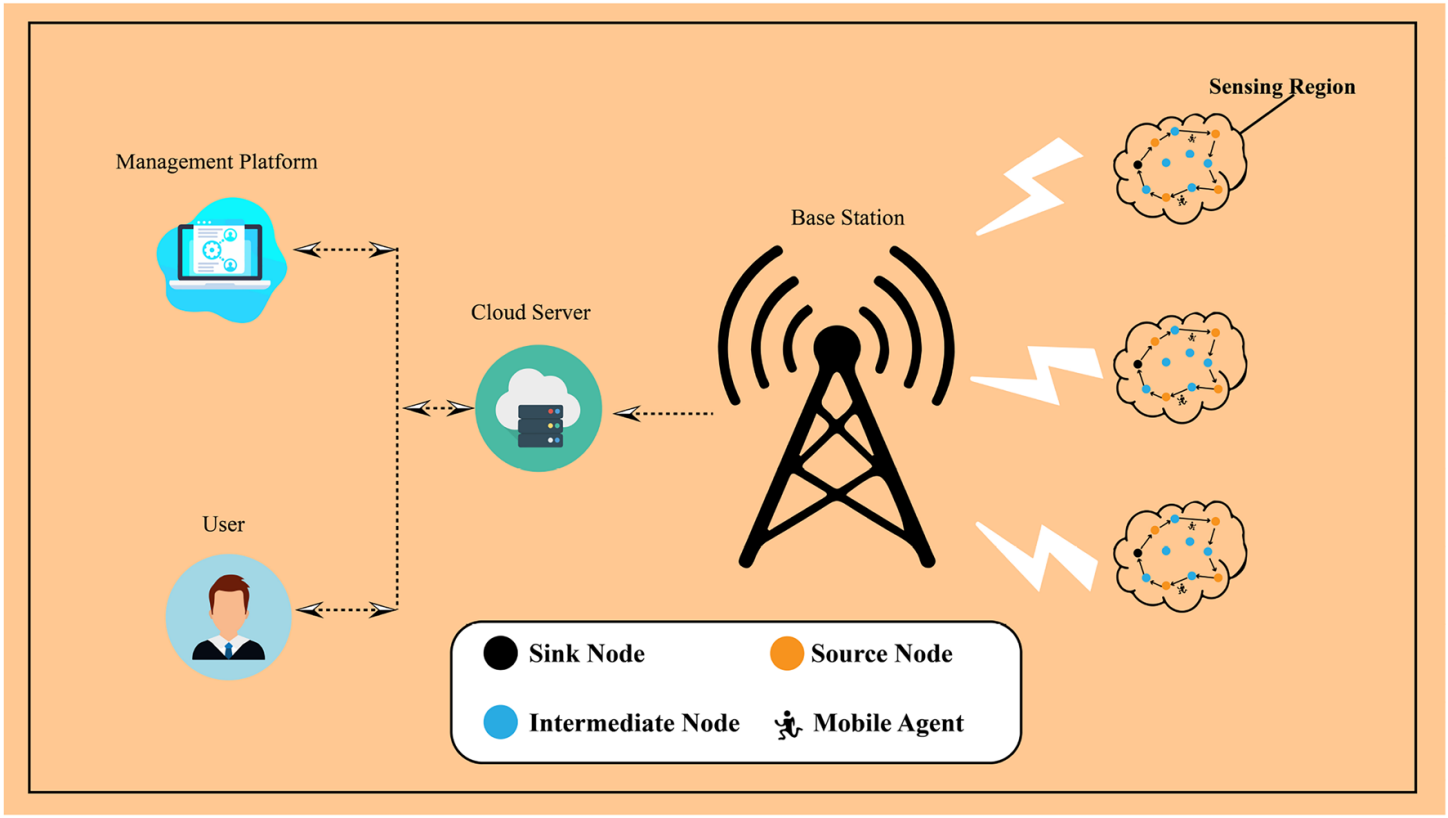
Architecture of air pollution monitoring system.

The architecture of an APMS is shown in Fig. 1. The mobile agent in wsn is responsible for gathering data from multiple interconnected devices. MA emphasizes the collection of data from sensors located at each observation point for air pollution. The two primary tasks involved in this process are the retrieval of data from sensors and the creation of middleware that utilities mobile agents to transmit the data to the sink node. This middleware is known as Eagilla^56^ and is agent-based in nature. The process of collecting and aggregating data occurs in this domain because of the abundance of available data sources. The sink node is responsible for pre-processing and filtering tasks because it contains superfluous data. The transmission of data from the sink node to the cloud requires the implementation of an intermediary communication layer. This layer comprises various communication technologies such as LTE and Wi-Fi. This is the location where all the sensor data are transmitted for cloud-based data processing. This cloud infrastructure can be utilized as gateways capable of real-time processing. Cloud computing has the potential to enhance the cloud latency. This location facilitates real-time decision making. Data management constitutes the fundamental layer responsible for information storage and analysis. The integration of multiple external technologies is feasible in this context, as expeditious data processing is imperative for analytical purposes^57^ . The data acquisition stratum of a smart urban environment aggregates information from a multitude of interconnected devices such as sensors.

Bayesian regression produces additional instances DF(z;$\Theta$) is given as

4 $$\begin{aligned} DF(z; \Theta ) = { \frac{1}{m} { {\sum _{j=1}^{m}} {\mathbb {N}} ({\Delta }_r(\Theta ) \ge z) } } \end{aligned}$$

As demonstrated in Eq. (4), forecasts ${\Delta }_r(\Theta )$, the noise has a specific probability distribution N, the objective of signal z and m size of the population. Distributed data estimation methods can be employed using the distance measures DI(R) as follows:

5 $$\begin{aligned} DI(R) = { \frac{{\sum _{j=1}^{} {u_j} {x_j}}}{ {\sum _{j=1}^{} {u_j}}}} \hspace{5mm} where \hspace{1mm}{u_j} = g(h(R,Q_j)) \end{aligned}$$

As illustrated in Eq. (5), a weighted average is employed, to estimate the g value for a given query point ${u_j}$, where a distance-based function ${x_j}$ determines the weights. The observed function demonstrated a gradual decrease in the range between R and $Q_j$. This location serves as an educational resource for individuals seeking to expand their knowledge on pollution and determine actionable steps towards its mitigation. A web-based APM service (P) is designed to provide users with the ability to track various air pollution metrics.

6 $$\begin{aligned} S(W) = \min (f_{ab}) \times h(R,Q_j) \end{aligned}$$

The user interface is denoted by $f_{ab}$, whereas the device status is indicated by $h(R,Q_j)$ in Eq. (6). In addition, the real-time data storage on the server is represented by variable j. Air pollution has no discernible effect on individuals who are in good health. Variable B (x; $\Theta$) is defined as the air quality level during the test days given that it is at a sufficiently high level.

7 $$\begin{aligned} B(x; \Theta ) = { C(x; \Theta ) {w_r} - {m}_{r} } \end{aligned}$$

As illustrated in Eq. (7), a current services C(x; $\Theta$ ) s utilized in order to assess the strength of living organisms ${w_r}$, and monitor weather conditions ${m}_{r}$.

A comprehensive web-based platform has been developed to collect, organize, store, and retrieve data and knowledge from various sources in real time. This platform allows data modification, analysis, and visualization, utilizing online portals and SMS notifications. The GPS technology integrated into the 4G model enables real-time surveillance and seamless connectivity to LTE networks.

## Methodology

To address the limitations of existing routing algorithms, especially in terms of adaptiveness and energy efficiency in dynamic wsn environments. this study introduces the Fuzzy Logic-enhanced Particle Swarm Optimization (FLPSO). The proposed method aims to integrate fuzzy rule-based intelligence with swarm optimization to enhance real-time decision-making, minimize energy usage, and reduce latency. The following section elaborates on the structural and algorithmic design of FLPSO.

As noted earlier, the proposed FLPSO method utilizes three input parameters. To mitigate the problem of fuzzy rule explosion, the number of input variables was limited to three. This is because increasing the number of inputs in the fuzzy logic model (FLM) leads to a more complex rule base. Alternatively, approaches like the Hierarchical Fuzzy System (HFS)^67^ have been developed to reduce the size of the rule base without compromising the system’s accuracy.

### Initialization

The FLPSO framework incorporates a diverse set of swarm sensor particles. Each sensor particle, denoted as ${P}_{i}$, operates within a physical coordinate system, where D represents the set of varying positional dimensions^68^. The ${i}^{th}$ sensor particle ${P}_{i}$ distinct position is illustrated in Eq. (8).

8 $$\begin{aligned} {P}_{i}=[{P}_{i,1},{P}_{i,2}\ldots \ldots ..{P}_{i,D}] \end{aligned}$$

Each sensor particle ${P}_{i,1}$ occupies a position in the network space, represented by the coordinates ($(x_{i,1},y_{i,1})$) as illustration in Eq. (9).

9 $$\begin{aligned} {\textrm{P}}_{\textrm{i}}=[(\textrm{x}_{\textrm{i,1,}} \varvec{y}_{\textbf{i,1,}}\mathbf {)} \textbf{,}{ \varvec{(x}}_{\textbf{i,2,}} \varvec{y}_{\textbf{i,2}}\mathbf {)} \ldots \ldots ..{(x}_{i,D,}y_{i,D,})] \end{aligned}$$

where i represents the number of sensors.

### Fuzzy logic input metrics

Normalization across deployments. To ensure portability across cities, RE, DC, and NN are normalized using a short calibration run to estimate deployment percentiles. All membership functions are then defined on the [0,1] scale, enabling the same rule base (2) to operate without change while only rescaling breakpoints to local operating ranges.

#### Node’s remaining energy

Represents the residual power of a node, computed in Eq. (10).

10 $$\begin{aligned} RE\left( {{P}_{i} }\right) =\frac{E_{depleted}}{E_{initial}} \end{aligned}$$

#### Distance to the source node

Denotes the Euclidean distance between a candidate node and the source node^69^ is provided in Eq. (11).

11 $$\begin{aligned} D(C)=\sqrt{(P_{x}-C_{x})+(P_{y}-C_{y})} \end{aligned}$$

where (${P_{x}}$ and ${P_{y}}$ ) denote the coordinates of the next source node and (${C_{x}}$ ) represent the coordinates of each CN.

#### Number of neighbors

Reflects nearby nodes within transmission range, influencing route redundancy and stability. A higher neighbor count reduces risk of node isolation and enhances routing efficiency^11^.

### FLM design in PSO

**Fig. 2:**
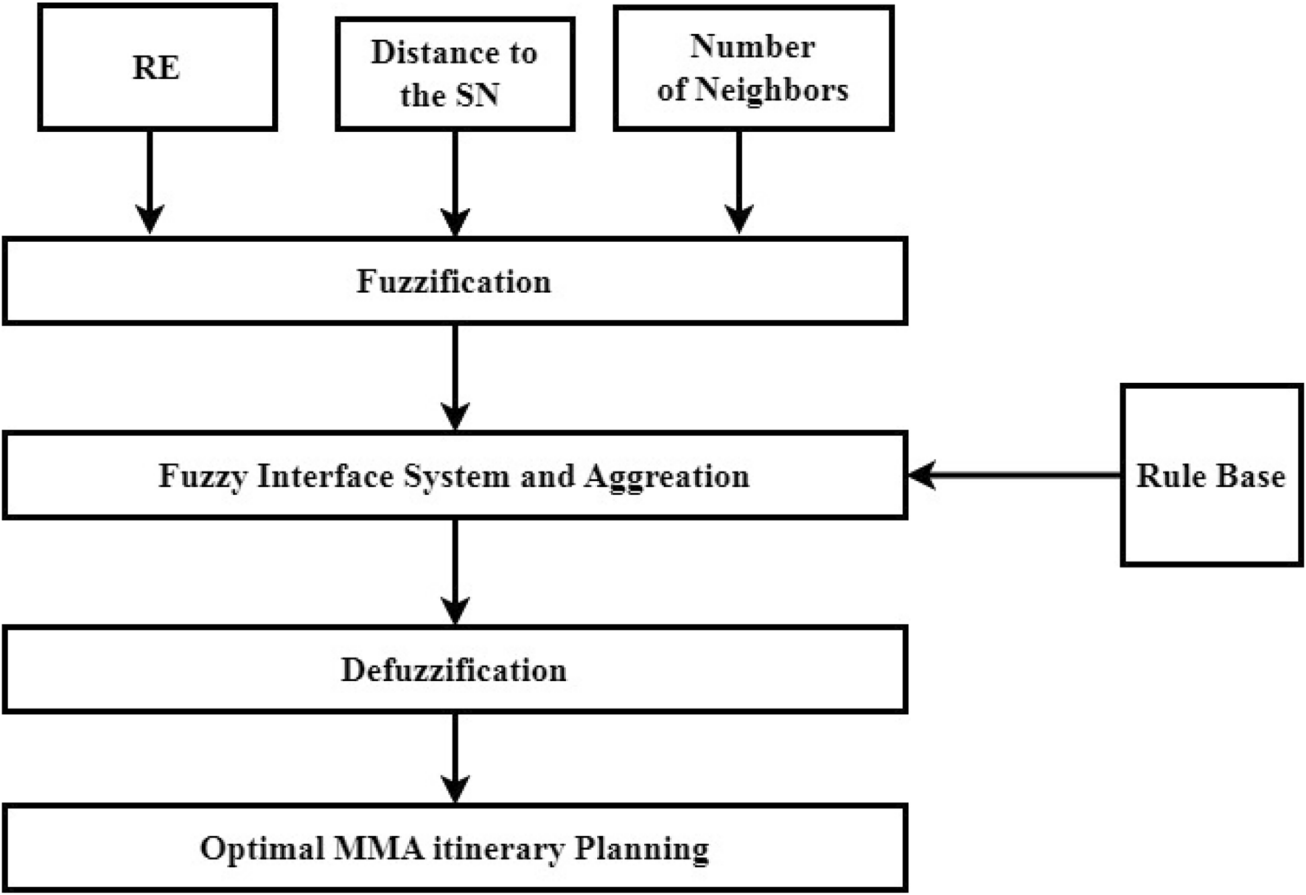
FLPSO inference system.

The main goal of this investigation is to find the best possible routing path for mobile agents by applying the Particle Swarm Optimization(PSO). At every next-hop decision, the proposed FLPSO method considers three significant parameters of the path the mobile agent is moving along that need to be evaluated. The fuzzy logic model (FLM) displayed in Fig. 2 . FLPSO considers the conditional probabilities relevant to each possible node to decide the best next hop for the movement of the agents^70,71^. Each of these four phases is described further below.

#### Fuzzification

At this time specific input variables for each candidate node such as residual energy, distance and number of neighbor are collected as reported in Fig. 2. In a fuzzy set, the role of membership function and linguistic parameters is important to represent the real conditions as well as possible. While traditional sets contain only numerical values, fuzzy sets are represented using language or expressions to carry their meaning. Linguistic inputs were interpreted using triangular and trapezoidal membership functions within the FLPSO approach in this analysis. Regarding Eq. (12) and Eq. (13), triangular and trapezoidal membership functions were chosen for their computational simplicity and good performance in real time applications, being applicable to both input and output fuzzy variables^72,73^.

12 $$\begin{aligned} \mu _{C1}\left( {w }\right) ={\left\{ \begin{array}{ll} 0 & w\le d1\\ \frac{w-d1}{e1-d1} & d1\le w\le e1\\[3pt] \frac{f1-w}{f1-e1} & e1\le w\le f1\\ 0 & f1\le w\\ \end{array}\right. } \end{aligned}$$

*d*1, *e*1 and *f*1 are parameters that define the geometry and placement of the triangular shape. Eq. (13) represents the standard form of the trapezoidal membership function.

13 $$\begin{aligned} \mu _{C2}\left( {w }\right) ={\left\{ \begin{array}{ll} 0 & w\le d2 \\ \frac{w-d2}{e2-d2} & e2\le w\le f2\\ 1 & e2 \le w\le f2 \\ \frac{f2-w}{f2-e2} & f2\le w\le g2 \\ 0 & g2\le w\\ \end{array}\right. } \end{aligned}$$

The parameters *d*2, *e*2, *f*2 and *d*2 characterize the structure and location of the trapezoidal function.

**Fig. 3:**
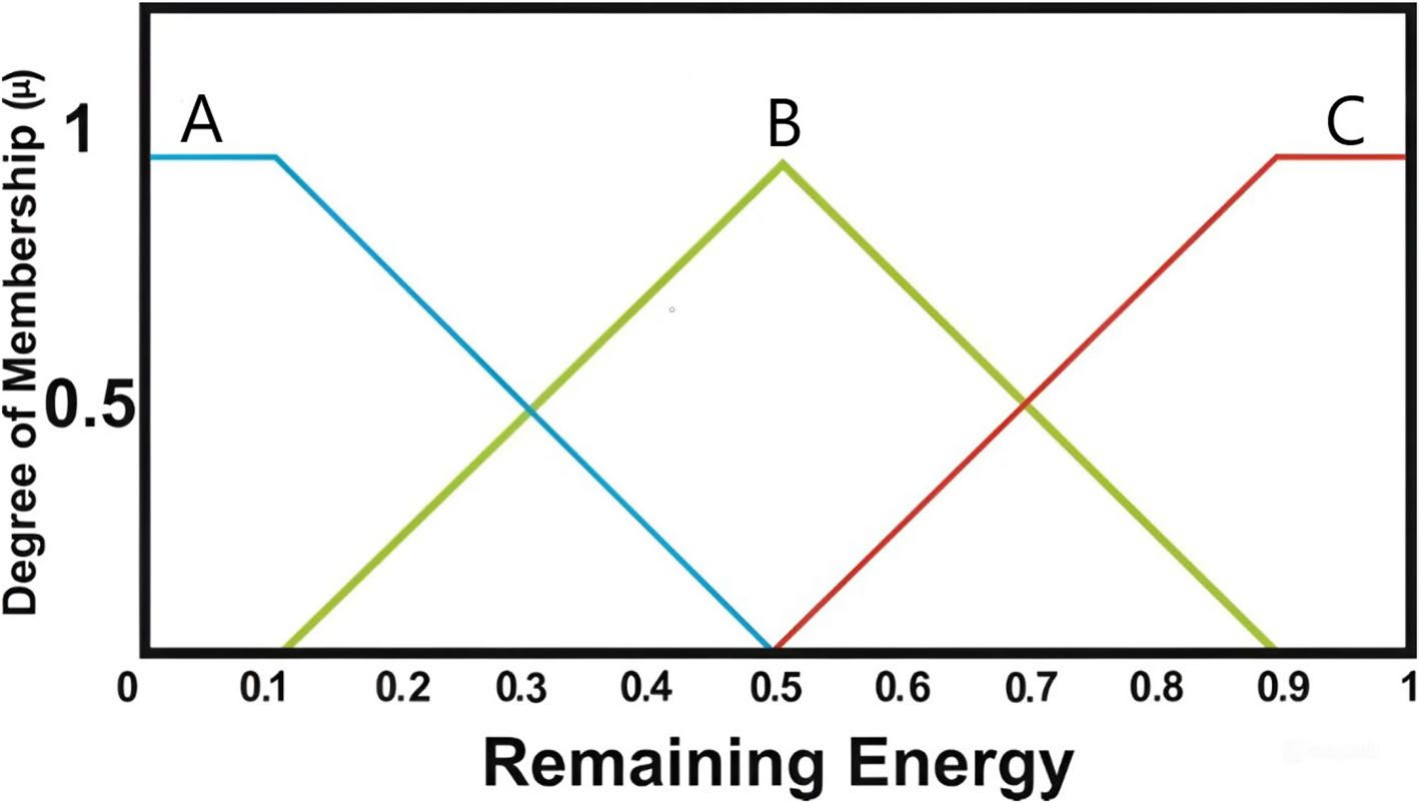
RE degree of membership function.

This is graphically depicted in Fig. 3, which presents the membership function associated to the residual energy parameter. The linguistic variables considered in this setting are A, B, and C. Membership values for these linguistic categories are defined within the following intervals: A from 0 to 0.1, B is 0.5, and C: 0.9 – 1.

**Fig. 4:**
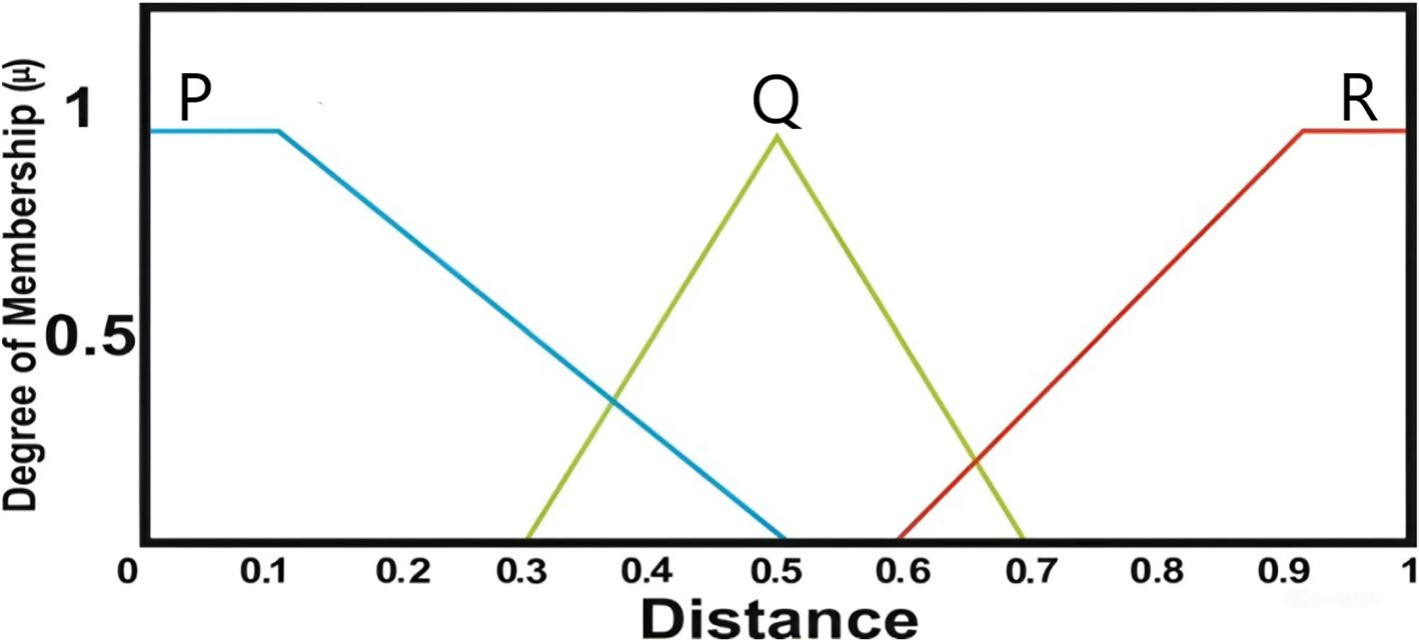
Distance degree of membership function.

The linguistic variables P, Q and R are modeled using triangular membership functions, each with a membership value ranging from 0 to 1. As illustrated in Fig. 4, the P variable spans values from 0 to 0.5, while R ranges from 0.6 to 1. The Q variable is centered at 0.5.

**Fig. 5:**
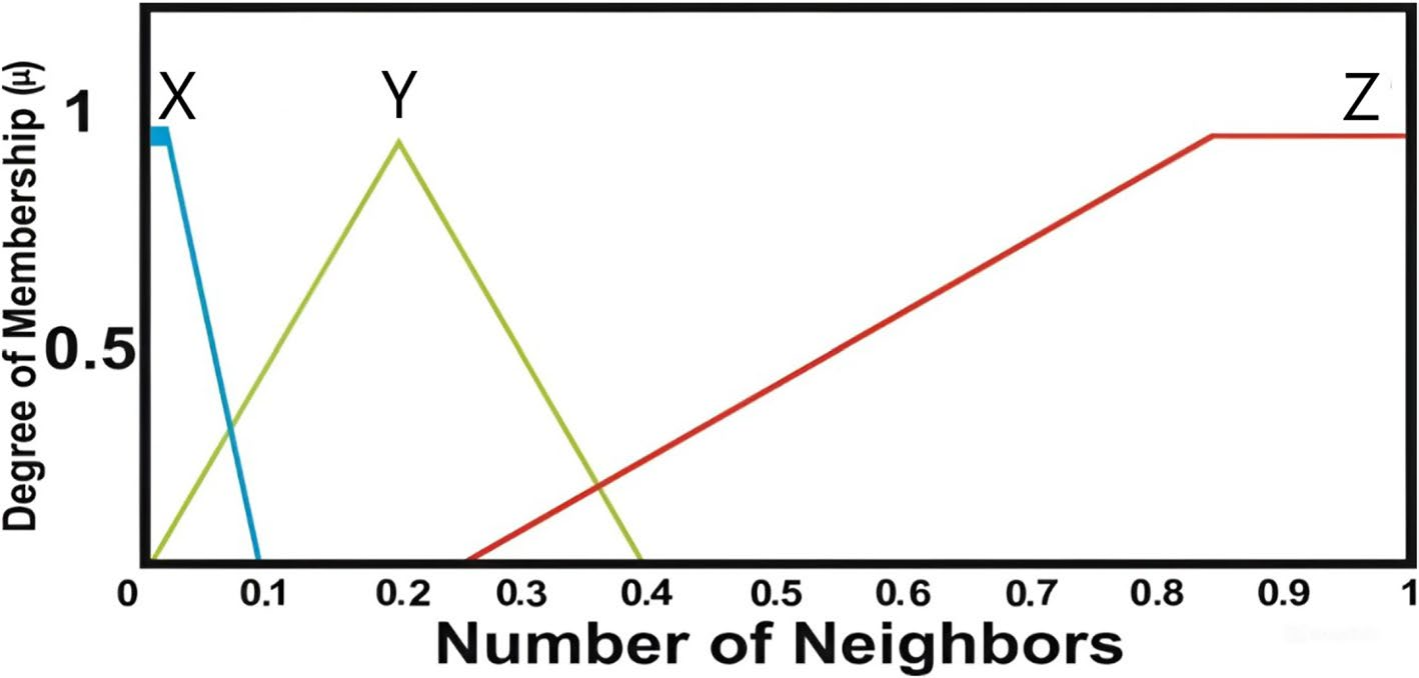
Node neighbor degree of membership function.

Figure 5 presents the membership function corresponding to the number of neighbors. The linguistic variables used in this context are X, Y, and Z. The X variable covers the range from 0 to 0.1, Z spans from 0.3 to 1, and Y is centered around 0.2.

#### Rule evaluation

The fuzzy output set is derived using a series of IF-THEN rules, which are formulated as conditional statements based on the membership values of the input variables. The fuzzy logical operator (AND) is employed to evaluate the conjunction of multiple input conditions within each rule. The complete set of IF-THEN rules is presented in table 2.

**Table 2:** Fuzzy IF-THEN rules in FLPSO approach.

Remaining Energy	Distance	Node Neighbor	probability
A	P	X	V-Low
A	P	Y	V-Low
A	P	Z	V-Low
A	Q	X	V-Low
A	Q	Y	V-Low
A	Q	Z	Low
A	R	X	V-Low
A	R	Y	Low
A	R	Z	L-Low
B	P	X	V-Low
B	P	Y	Low
B	P	Z	Low
B	Q	X	V-Medium
B	Q	Y	Medium
B	Q	Z	L-Medium
B	R	X	V-Medium
B	R	Y	Medium
B	R	Z	L-Medium
C	P	X	V-Low
C	P	Y	Low
C	P	Z	L-Low
C	Q	X	L-High
C	Q	Y	High
C	Q	Z	High
C	R	X	V-High
C	R	Y	V-High
C	R	Z	V-High

#### Fuzzy inference system and aggregation

At this phase, input observations are used to combine fuzzy rules and generate a unified fuzzy result. The Mamdani inference technique^74^ was selected for its ease of implementation in deriving output values across various possible nodes.

#### Defuzzification

Once the outputs of all fuzzy rules have been aggregated, the defuzzification process is applied to generate a crisp output. In this study, the Center of Area (CoA) method^75^. The commonly used defuzzification technique–was employed to compute the final crisp value. The corresponding calculation is presented in Eq. (14).

14 $$\begin{aligned} CoA\left( {w }\right) =\frac{\int {\mu _{c}(w)} wdw}{\int {\mu _{c}(w)} dw} \end{aligned}$$

**Fig. 6:**
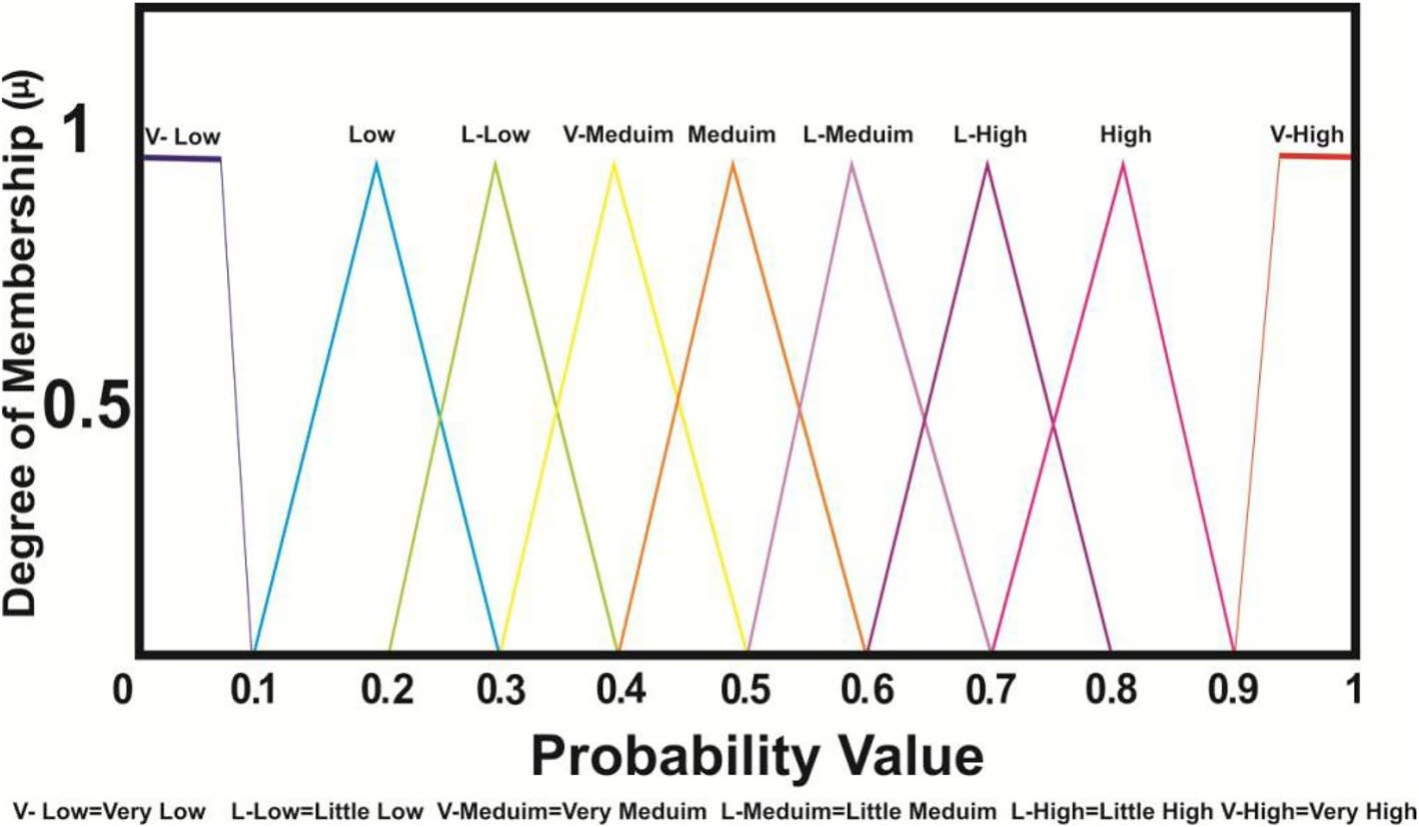
Membership of the probability value.

Here, w represents the output obtained after defuzzification and $\mu c(w)$ denotes the aggregated membership function. The probability range was partitioned into nine linguistic levels at intervals of 2.5 cm, as depicted in Fig. 6. For a CN, a higher defuzzified output value increases the likelihood of it being selected as the next migration hop for the mobile agent.

### Velocity and practice position update

In each iteration t, the sensor particles $P_{i}$ update their individual best-known positions $P_{best}$ and $G_{best}$.. Each particle moves toward $G_{best}$, by updating its velocity $Vel_{i,d}$ and positions $P_{i,d}$ during each iteration. The calculation for the velocity $Vel_{i,d}$ is provided in Eq. (15).

15 $$\begin{aligned}&{Vel}_{i,d}\left( {t }\right) =w\times {Vel}_{i,d}\left( {t-1 }\right) +c_{1}\times {rand}_{1} \nonumber \\&\times ({P}_{Pbest_{i,d}}-{P}_{i,d}\left( {t-1 }\right) +c_{2}\times {rand}_{2} \nonumber \\&\times ({P}_{Gbest}-{P}_{i,d}\left( {t-1 }\right) ) \end{aligned}$$

where $c_{1} \hspace{1mm} and \hspace{1mm} c_{2}$ represent the acceleration coefficients, which are constant values. w denotes the inertial weight. The$rand_1 \hspace{1mm} and \hspace{1mm} rand_2$ are random values uniformly distributed between 0 and 1. The position update of $P_{i}$ is given by Eq. (16).

16 $$\begin{aligned} {P}_{i,d}\left( {t }\right) ={P}_{i,d}\left( {t-1 }\right) +{Vel}_{i,d}\left( {t-1 }\right) \end{aligned}$$

### Fitness function evaluation

After each iteration, the sensor particles update their positions, and the fitness function^50^ is evaluated to determine whether a particle has achieved the global best position Gbest. The fitness function evaluation is conducted using the formulas provided in Eq. (17) and Eq. (18).

17 $$\begin{aligned} P_{best_{i}}=&{\left\{ \begin{array}{ll} {P}_{i} & if\left( {Fitness\left( {{P}_{i} }\right) < Fitness\left( {P_{best_{i}} }\right) }\right) \\ P_{best_{i}} & Otherwise\\ \end{array}\right. } \end{aligned}$$

18 $$\begin{aligned} G_{best}=&{\left\{ \begin{array}{ll} {P}_{i} & if\left( {Fitness\left( {{P}_{i} }\right) < Fitness\left( {G_{best} }\right) }\right) \\ G_{best} & Otherwise\\ \end{array}\right. } \end{aligned}$$

###  Fuzzy logic based particle swarm optimization MIP

**Fig. 7:**
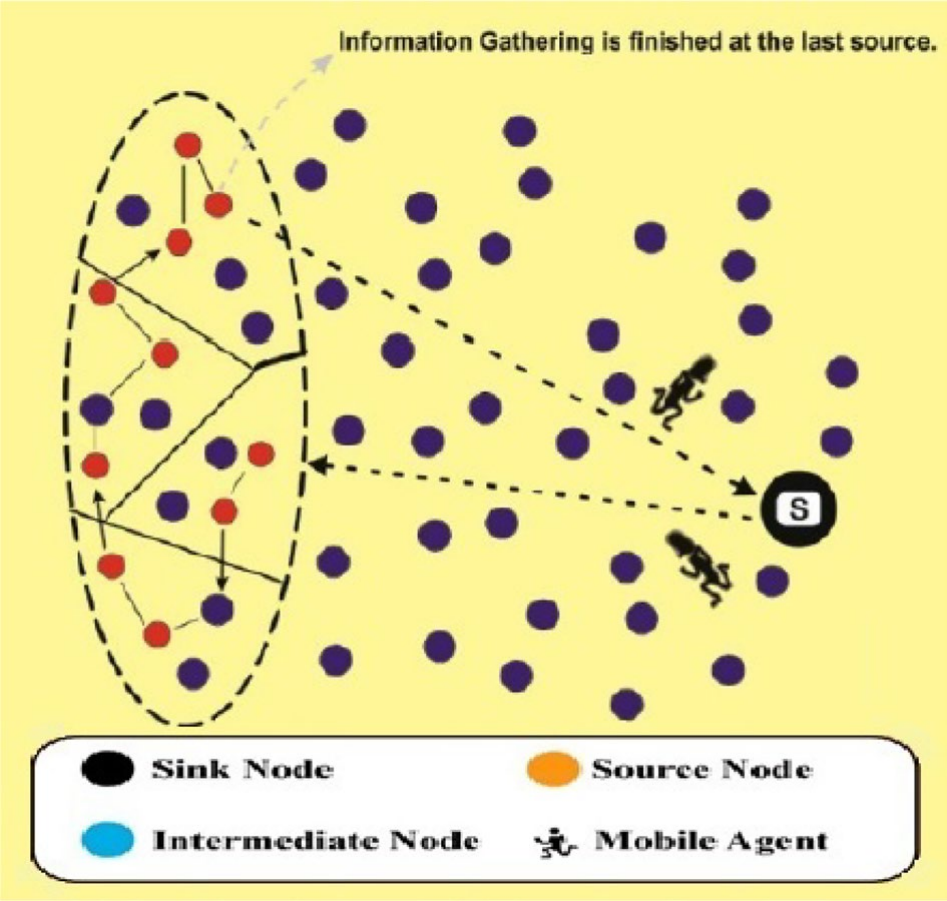
FLPSOMIP in WSN.

The FLPSO technique, which is a sophisticated computational framework based on the FLM, employs decision-making instruments to enhance networks efficiency^58^ . The FLPSO technique comprises three distinct stages. The initial phase functions in the following manner: selecting an optimal sink node and determining an accurate cluster size are crucial for achieving an equal load distribution during processing. The second step involves utilizing FLM to calculate the likelihood of nodes based on input parameters such as the remaining energy (RE) of the node, its distance from the source node (DC), and its number of neighbors. The resulting output characteristics are restricted by the probability of forming specifier paths to the MAs^59^. Third, each MA traverse the network in accordance with predetermined paths prior to commencing the process of acquiring information, as depicted in Fig. 7. The generated routes aim to encompass all nodes within the network. The sink node assumes the responsibility of allocating MAs to a specific group to gather the data. The MA individually gathers information from the assigned groups. To streamline local interactions and minimise reporting delays, we employed a variety of MAs. The methodology employed for determining the quantity of sensory data to be gathered by multi-agent systems is analogous to that outlined in references^60,61^.The pseudocode of the FLPSOMIP algorithm tailored for WSNs is presented in 1. The fuzzy logic rule evaluation incurs a computational time complexity of O($m \times k + n$) where m is the number of input metrics (e.g., residual energy, delay), k the number of membership functions per metric, and n the number of fuzzy rules. For our implementation, m=3, k=3, and n=27, leading to a lightweight inference process.

**Algorithm 1:**
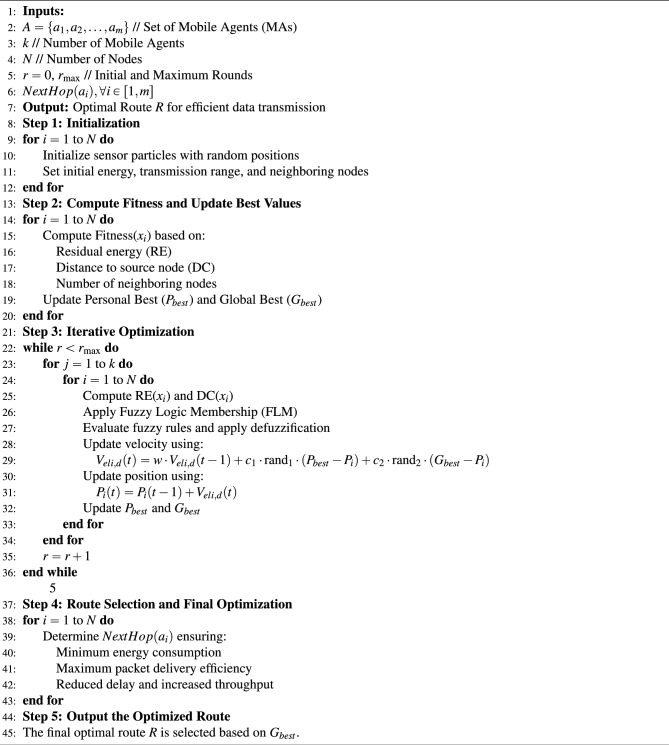
FLPSO Algorithm for Optimized Routing in Air Pollution Monitoring Systems.

### Congestion-aware FLPSO and hierarchical scaling

To extend FLPSO-AMPS beyond small deployments, we introduce scalability mechanisms. First, the fitness function is augmented with congestion-awareness, incorporating both queue length (q) and link utilization

19 $$\begin{aligned} Fitness = \alpha \cdot Energy + \beta \cdot Distance + \gamma \cdot Neighbors + \delta \cdot q + \epsilon \cdot u \end{aligned}$$

This ensures that next-hop decisions avoid overloaded nodes and links. Second, we adopt hierarchical clustering, with residual-energy-based cluster-head rotation and multiple sinks to reduce path length and contention. Third, mobile-agent itineraries are partitioned across clusters so that latency grows sublinearly with network size. These enhancements preserve energy efficiency while stabilizing throughput and packet delivery ratio in large-scale deployments.

### Tuning and sensitivity of fuzzy rules

The FLPSO-AMPS fuzzy controller employs triangular/trapezoidal membership functions for three network-centric inputs: remaining energy (RE), distance to source (DC), and number of neighbors (NN) (see Section 3.2). To generalize across cities with different densities and traffic dynamics, inputs are first normalized via deployment statistics. Let $x \in \{\text {RE}, \text {DC}, \text {NN}\}$ and let$([ \ell _x, u_x ])$ denote the empirical 5th–95th percentiles measured during a short calibration phase. We then scale $\tilde{x} = \frac{x - \ell _x}{u_x - \ell _x} \in [0,1],$ and clip values outside this range. Initial membership breakpoints ((*d*, *e*, *f*, *g*)) for each linguistic label are seeded from percentiles (e.g., 25%, 50%, 75%) of $\tilde{x}$.

We refine $(d,e,f,g)$ using a constrained lightweight optimizer (PSO/Bayesian search) that maximizes a cross-validated objective:

20 $$\begin{aligned} \max _{\theta } \; J(\theta ) = w_{1}\,\text {PDR} - w_{2}\,\text {Delay} - w_{3}\,\text {Energy} - w_{4}\,\text {PLR}, \end{aligned}$$

subject to monotonicity constraints that preserve interpretability: $\uparrow \text {RE} \;\Rightarrow \; \uparrow \text {output}, \quad \uparrow \text {DC} \;\Rightarrow \; \downarrow \text {output}, \quad \uparrow \text {NN} \;\Rightarrow \; \uparrow \text {output}.$

The fuzzy rule base remains fixed only membership parameters $\theta = (d,e,f,g)$ and input scalers are tuned, keeping per-hop inference complexity unchanged.

Robustness is quantified by two complementary analyses:

(i) Local one-at-a-time (OAT) perturbations of each membership breakpoint by $\pm \{10,20,30\}\%$, while tracking performance metrics $\{\text {PDR}, \text {Delay}, \text {Energy}, \text {PLR}\}$.
(ii) Global variance-based sensitivity analysis (Sobol indices) over the joint space of $\theta$ and traffic intensity $\lambda$.

We also evaluate context shifts (traffic-dominated vs. industrial-dominated vs. mixed corridors) by altering spatial load maps and link reliabilities. These studies show that the controller’s performance degrades gracefully under parameter drift and that the relative influence of RE, NN, and DC aligns with intuition across contexts.

## Data collection, experimental setup, and APMS analysis

###  Sensor deployment and data collection

**Fig. 8:**
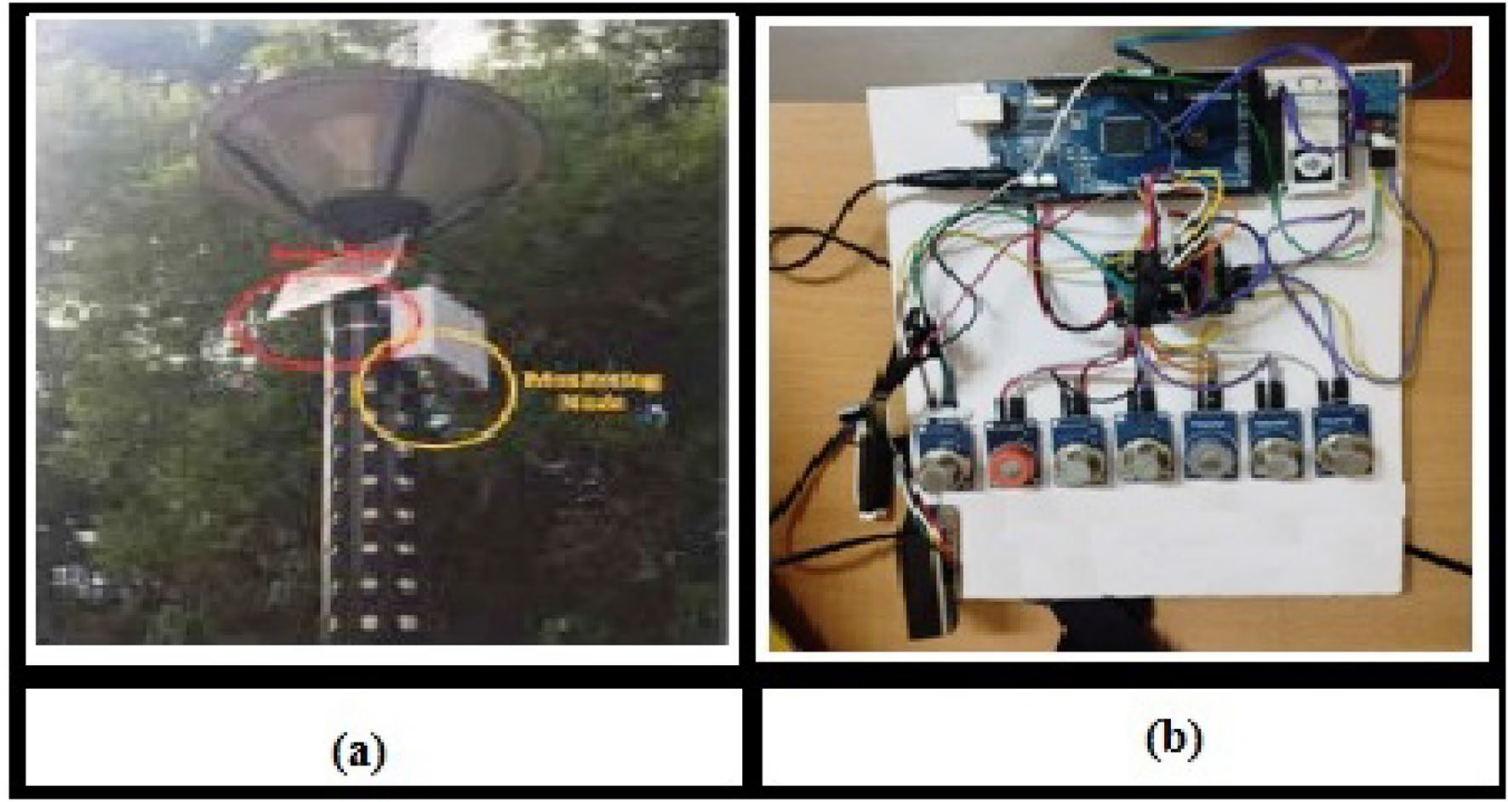
(**a**) Photo of the monitoring node. (**b**) low-cost sensors used in our Ballari campaign.

The ballari district is an excellent example of a neighborhood comprising detached homes, attached houses, and apartments. The air quality in this area is predominantly affected by factors such as the use of building components, industrial emissions of CFCs, trash burning, road dust,fine ash and agricultural waste. The high residential density in urban locations can lead to significant smoke nuisance for immediate neighbors^62^. Additionally, local air quality can deteriorate due to geographical and weather conditions, particularly on cold and windy days. Our campaign utilized sensory data collected between january 1, 2023, and april 30, 2023. We deployed thirty-three devices in various locations corresponding to the city ward shown in Fig. 8. The measurement analysis focused on an area of approximately 8 $KM^2$. The experiment aimed to assess the performance and accuracy of these cost-effective devices in the target area. Further experiments were conducted in the winter and summer of 2023 to evaluate the effectiveness of using the generated data by comparing it with the information provided by the pollution board and specific contaminants in the region^63^.

To validate our sensor measurements, we used the AQM station as the primary reference station. The measurement campaign occurred in the ballari district, adjacent to the hospet neighborhood, where the vijayanagara air quality station is situated. This station, located in the northern hospet, is approximately 3 km away from the city center. Ballari is renowned for its thermal power plant, industries, and iron ore mining, resulting in pollution from waste burning, coal, and wood and transportation. This motivated us to install affordable sensors and conduct a measurement campaign in the ballari district. The vijayanagara station, with its sampling intake positioned approximately 8 m above the ground, is an urbanized sensing station that monitors various aspects, such as greenery, roads, parking lots, and buildings. We collected data from the vijayanagara station to assess readings from our compact sensors. We computed the average of our sensor data as 1-hour averages, aligning them with the information gathered from the Vijayanagara station. We conducted a comparison between the readings obtained from our experiment and the data collected from vijayanagara station, thereby validating the performance of our measurements. the deployment of 33 sensors effectively ensures comprehensive spatial coverage and facilitates the detection of pollution patterns, conducting a formal cost-benefit analysis is crucial, particularly for scalability in Tier-2 cities. The initial deployment costs encompass sensor procurement, communication modules, and integration into the FLPSO-AMPS framework. Long-term maintenance costs, including periodic battery replacement, data transmission infrastructure upkeep, and sensor recalibration, can substantially affect the feasibility. In Tier-2 urban environments with limited municipal budgets, these recurring expenses present significant sustainability challenges. Additionally, environmental factors, such as dust accumulation, temperature drift, and humidity fluctuations, can lead to calibration errors over time, thereby impacting data fidelity. Addressing these issues necessitates the integration of self-calibration algorithms and robust error-compensation techniques. To address calibration drift and environmental interference common in low-cost sensors, each node incorporates a self-calibration routine. This includes periodic zero-point resets and offset estimation based on environmental baselines. Calibration flags are raised when deviations exceed thresholds, prompting remote updates or technician checks.

###  Low-cost sensors

To detect and monitor microparticle densities, we installed the Wisen ZH06-III laser dust sensor. This device provides accurate PM 2.5 and PM 10 particle mass readings, representing the proportion of nanoparticles with aerodynamics diameters less than 2.5 $\mu m$ and 10 $\mu m$ , respectively. It is accurate and highly reliable within a range of 0 - 10,000 particles/ $m^3$ . For the detection of VOCs, which are HC based substances with high vapor pressure, we utilized the Ogam Technology GSBT11-P110 VOC sensor module. This module can detect various VOCs, including formaldehyde, toluene, benzene, xylene, and organic solvents. Ammonia ($NH_3$) pollution is caused by the release of ammonia gas, a byproduct of both agriculture and industry. Common sources of ammonia pollution include decomposing agricultural slurry, fertilizer plants, and natural occurrences such as coal mine fires. To identify ammonia gas, we incorporated a Wisen ZP01-MP503 air quality detection module. This sensor module demonstrates sensitivity to ammonia as well as other substances, such as cigarette smoke, alcohol, and specific gases.

Carbon monoxide (CO) is a harmful by-product of the incomplete combustion of carbon-based fuels such as gas, oil, and coal. Inhalation of CO leads to its binding with hemoglobin in the bloodstream, thereby reducing oxygen transport and causing hypoxia. To monitor CO gas, we employed the Wisen ZE03 air quality electrochemical detection module, which is capable of detecting gases such as $CO_2$, $H_{2}$S, $NO_2$, $O_3$, $SO_2$, $CL_2$, HF, $H_2S$,$PH_3$, HCL, and other gases. For the detection of flammable gases, such as ethane, acetylene, and methanol, we utilized MQ-6 and ME4-ETO semiconductor sensors. A PAH500 Digital PAH sensor was employed to detect polycyclic aromatic hydrocarbons. Although $CO_2$ is not typically classified as an air contaminant, elevated levels of $CO_2$ in enclosed environments can lead to oxygen depletion and are considered pollutants. To measure and track $CO_2$ concentrations, we installed the MH-411D NDIR infrared $CO_2$ sensor module. Toxic metals, such as lead and mercury were measured using the Pb-023 lead sensor and the KY-017 mercury sensor module, respectively.

### Cost–benefit and maintenance at scale

A deployment cost–benefit analysis is essential for Tier-2 cities. The bill of materials for each sensor (dust, VOC, gas, metal modules, microcontroller, communication) is approximately USD 50–70 per node, making a 1,000-node deployment feasible under municipal budgets as shown in table 3. Maintenance overhead primarily arises from calibration drift and periodic battery replacement, which scale linearly with the number of nodes. To mitigate this, cluster-head rotation balances energy use, while over-the-air updates and self-calibration flags reduce technician visits. At scale, the combination of in-network aggregation and duty-cycling reduces data transmission costs by 30–40%, ensuring sustainability for long-term monitoring.

**Table 3:** Cost benefit analysis of FLPSO-AMPS deployment in tier-2 smart cities.

Item	Per-Node Cost (USD)	1k-Node Deployment (USD)	Maintenance Notes
PM2.5/PM10 sensor	20	20,000	Low-cost laser-based dust sensors
VOC sensor	15	15,000	Detects benzene, toluene, xylene
Gas sensors (CO, NH$\phantom{0}_3$, SO$\phantom{0}_2$)	20	20,000	Electrochemical, cross-sensitive
Metal sensors (Pb, Hg)	15	15,000	Toxic metal detection modules
Microcontroller (ESP32)	10	10,000	Low-power dual-core MCU
Communication module	10	10,000	WiFi/LoRa modules
Annual calibration	5	5,000	Annual recalibration required
Battery replacement	3	3,000	Replaceable every 18–24 months

### Reliability mechanisms for low-cost sensors

Ensuring long-term reliability requires compensating for the inherent limitations of low-cost sensor modules. Three mechanisms are integrated into FLPSO-AMPS deployments:

1. Self-calibration: Each node performs scheduled baseline checks against ambient conditions and, when available, co-located reference monitors. Drift is quantified and offsets are corrected in-situ.
2. Sensor fusion: Measurements from multiple pollutant modules (e.g., particulate, VOC, and gas sensors) are aggregated using weighted averaging and correlation checks, which help detect anomalies and reduce cross-sensitivity.
3. ML-based error correction: Lightweight regression models, trained during initial collocation campaigns with reference stations, correct systematic biases. The corrected values are applied in-field with negligible computational overhead on ESP32-class MCUs. These measures significantly improve consistency over extended deployments, reducing the mean absolute error by  15–20% compared with uncorrected readings.

###  Analysis of AQI using recorded data

To track the state of air in ballari city, 33 APMS instruments were placed. After installing The FLPSO-APMS, a cloud-based web portal was activated to analyze the gathered information and provide visual representations of air quality on the application^64,65^. The data from each device were categorized by the area and device ID. The cloud produced reports that could be extracted for analysis for the current set of saved data with measured times. The data were colour-coded and visualised depending on the air quality at the time. The device’s hue turned yellow or red whenever air quality was medium or poor, and the warning feature was activated. A mobile application was made available for remote air quality monitoring^66^.

###  Data integration and visualization

**Fig. 9:**
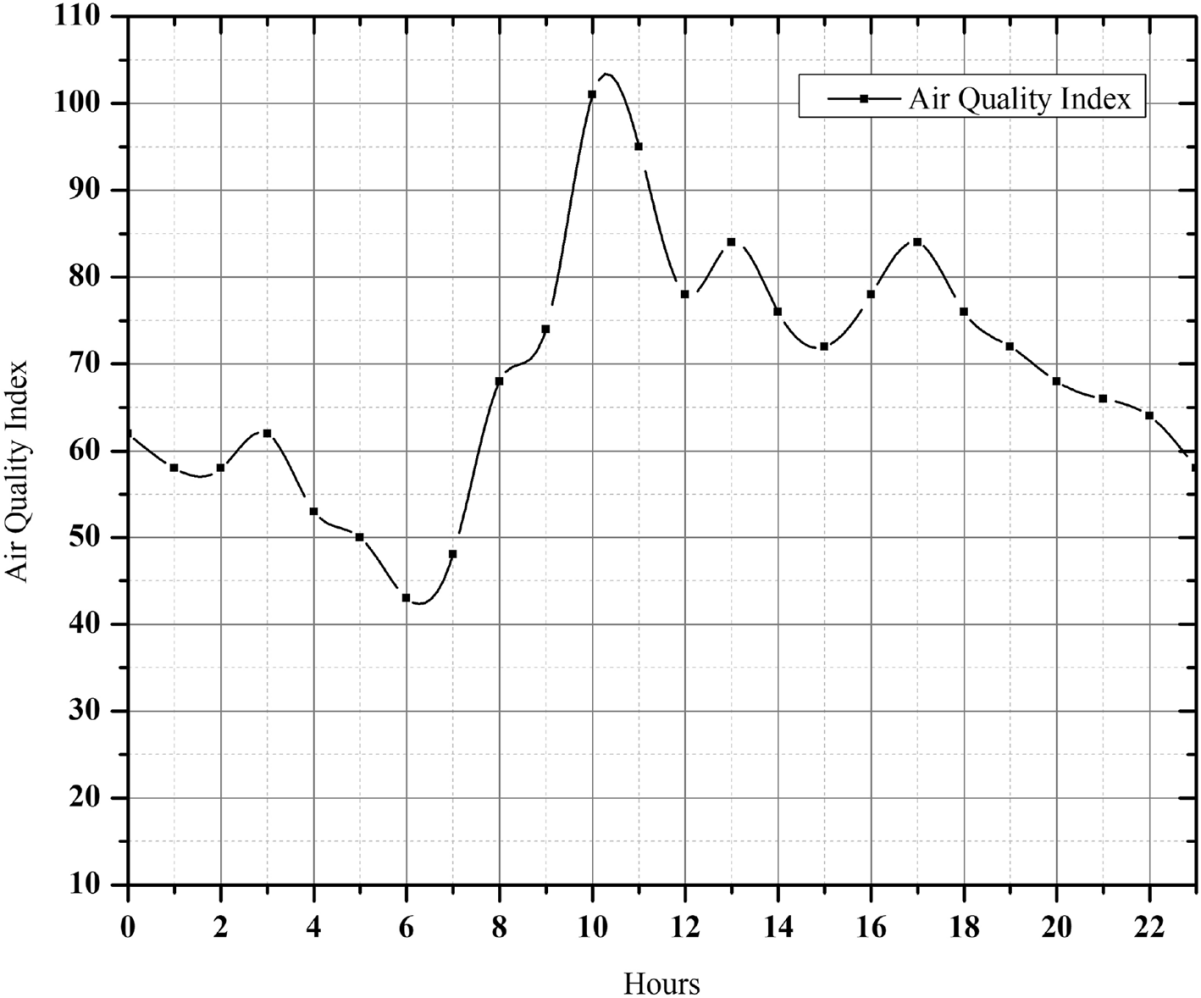
AQI values of a day.

The critical aspect of our research lies in providing a daily air pollution profile specifically for ballari. This achievement is made possible by the use of portable and affordable sensors^67,68^ . Likewise, the identification and analysis of air pollution hotspot profiles holds significant importance in the development of intervention strategies within a given region. Indeed, these findings have the potential to inform decisions pertaining to adjustments in road traffic infrastructure or the development of new green spaces^69,70^. The variables of primary interest for the detection of air pollution include AQI, PM2.5, PM10, CO, $NO_2$, $SO_2$, and $O_3$. The emission of the air quality index tended to rise during peak traffic hours, specifically from 8:00 a.m. to 10:30 a.m. in the morning and from 4:30 p.m. to 7:00 p.m. in the afternoon Fig. 9. This increase can be attributed to the heightened use of vehicles by individuals commuting to and from their workplaces^71–73^. An increase in the AQI to 101 specifically due to the presence of PM10 particles, is attributed to the emission of dust particles from sources such as heavy traffic, smoke, and construction sites. These particulate matter pollutants have the potential to induce various health effects, including eye irritation, respiratory tract infections, and lung cancer, as reported by the CDC. Similarly, fine particulate matter, specifically PM2.5, was predominantly generated as a result of combustion emissions. These particles pose an even greater risk as they possess dimensions smaller than 2.5 $\mu$m, enabling them to penetrate the respiratory system and potentially enter the bloodstream.

**Fig. 10:**
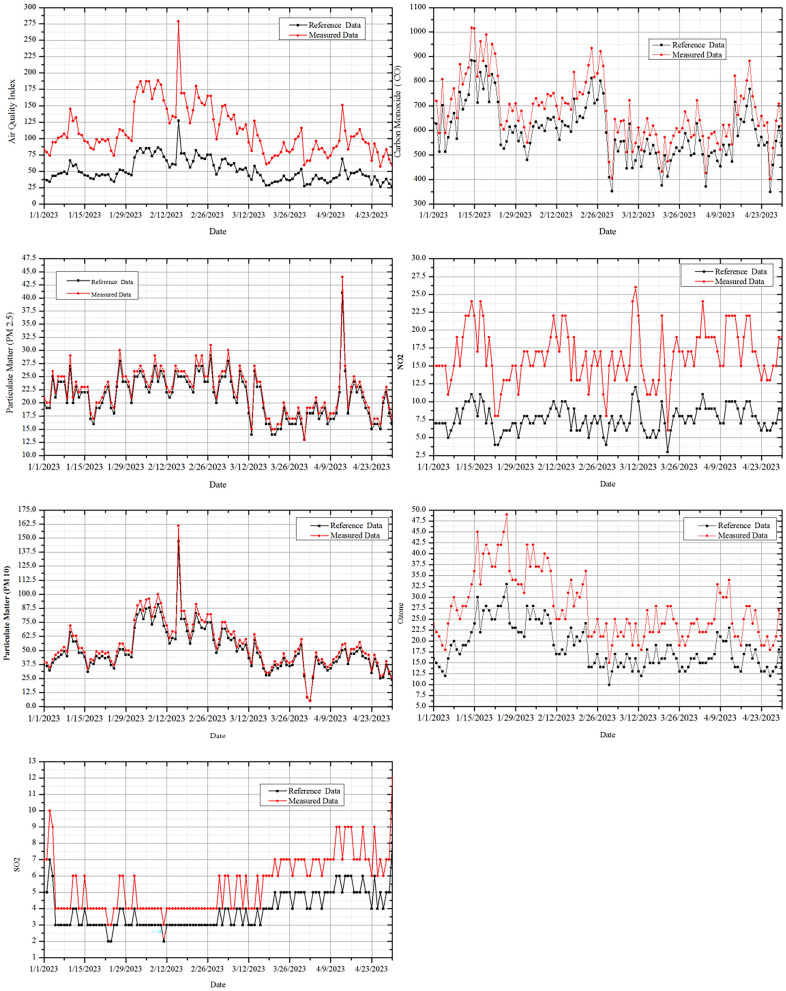
Reference data v/s measured value of AQI, CO levels, PM2.5, PM10, $SO_2$ levels, $NO_2$ levels, and Ozone levels.

For the time period from january 1, 2023, to april 1, 2023, the provided data represent the reference and measured air quality parameters. The characteristics are presented in Fig. 10 and comprise the AQI, CO levels, PM2.5, PM10, $SO_2$ levels, $NO_2$ levels, and ozone levels. The measured data represent the actual values recorded during the specified period, whereas the reference data served as a baseline for comparison. The data were displayed daily.

The AQI values of the measured data, which range from 27 to 189, showed a range of air quality from healthy to unhealthy. Carbon monoxide concentrations ranged from 352 to 1017 parts per million (ppm), with higher values indicating higher levels of CO pollution. PM2.5 concentrations vary from 13 to 30 g/$m^3$, whereas PM10 concentrations range from 8 to 161 g/$m^3$. While $NO_2$ levels vary from 4 to 26 g/$m^3$, $SO_2$ levels are between 2 and 10 g/$m^3$. The ozone concentration varied from 10 to 49 g/$m^3$. Overall, the information points to variations in air quality metrics over the course of the time period, with some days having higher pollution levels than others. A more thorough analysis of the data can reveal insights into certain trends or patterns and assist in identifying times when air quality has improved or declined^74,75^.

**Fig. 11:**
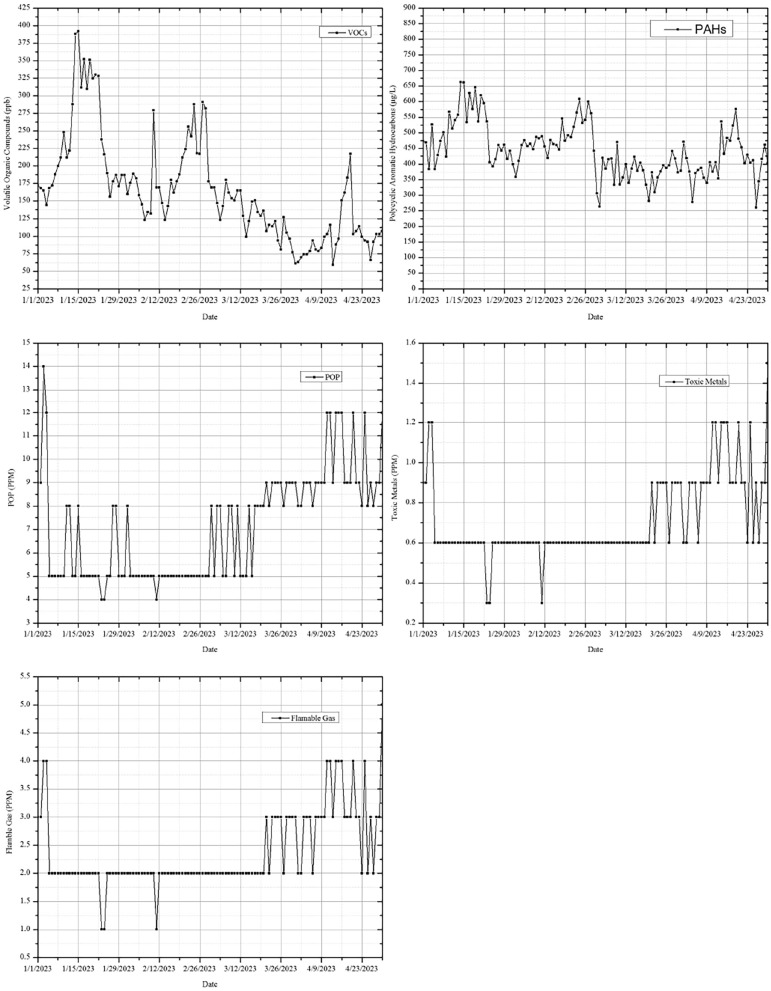
AQI parameters like VOC, POP, PAH, flammable gas, and toxic metals.

Information on air quality is gathered and examined using FLPSO-AMPS system. For the demonstration, only data from January 1, 2023, through April 30, 2023, is used. Figure 11 displays statistics on the air quality, including the VOC, POP, PAH, flammable gas, and dangerous metals^76–82^.

### Security considerations for FLPSO-AMPS

Environmental monitoring networks are highly sensitive because falsified data can distort public health responses. In FLPSO-AMPS, three classes of attacks are particularly relevant data spoofing (injecting false pollutant levels), packet tampering/replay (altering or re-using old measurements) and routing disruption (sinkhole or selective forwarding). To counter these threats on resource-constrained sensor nodes, several lightweight protocols are viable:

1. Symmetric cryptography with integrity checks: AES-CCM and HMAC-SHA-256 provide confidentiality and authenticity with <1 ms processing overhead on ESP32-class devices.
2. Reputation-based filtering: cluster-heads monitor packet consistency and penalize anomalous nodes, limiting insider threats.
3. Blockchain-inspired edge logging: sinks maintain an append-only ledger of received values and routing itineraries, ensuring auditability and preventing retroactive tampering.
4. Elliptic curve key management: ECC-based key refresh schemes provide forward secrecy with minimal key size overhead compared to RSA.

These mechanisms can be integrated incrementally, balancing energy cost against resilience, and collectively strengthen the trustworthiness of pollutant data.

## Simulation, results and discussion

This section outlines the simulation environment, the benchmarks employed, the state-of-the-art methods used for performance comparison, and the statistical analysis conducted.

###  Simulation setup

The simulations were conducted using MATLAB R2019a. The proposed FLPSO method was validated by deploying 100 wireless sensor nodes in a landscape measuring 1000 x 1000 m using MATLAB. The simulation parameters are presented in table 4.

**Table 4:** Simulation setup details.

Parameter	Value
Deployment Area	$1000 \times 1000$ m^2^
Input Data Type	Air pollution metrics
Total Sensor Nodes	1000
Initial Energy per Node	2 J
Range of Source Nodes	10–100
Communication Range	60 m
Raw Data Volume	2048 bits
Mobile Agent (MA) Code Size	1024 bits
MA Access Latency	10 ms
Data Compression Ratio	0.8
Aggregation Efficiency	0.8
Processing Speed	50 Mbps
Simulation Duration	20 rounds
Performance Metrics	TE, PDR, EDP, PLR, Throughput
Swarm Size	30
Acceleration Coefficients $C_1$, $C_2$	1.4495 each
Inertia Weight Range (*w*)	0.4 to 0.9

###  Performance metrics

**Fig. 12:**
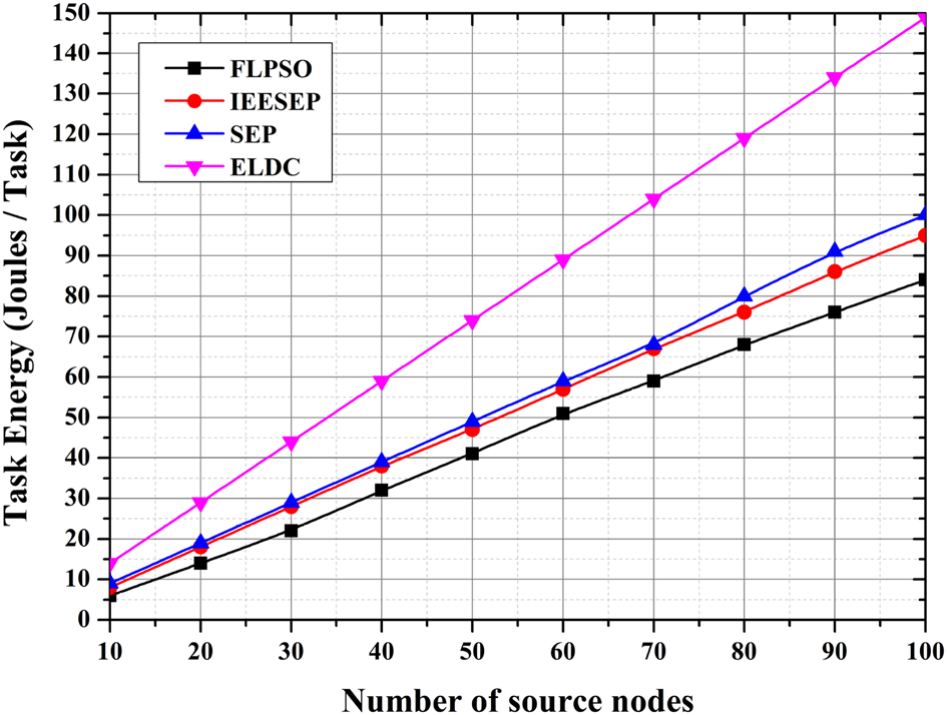
The impact of source node on task energy.

**Fig. 13:**
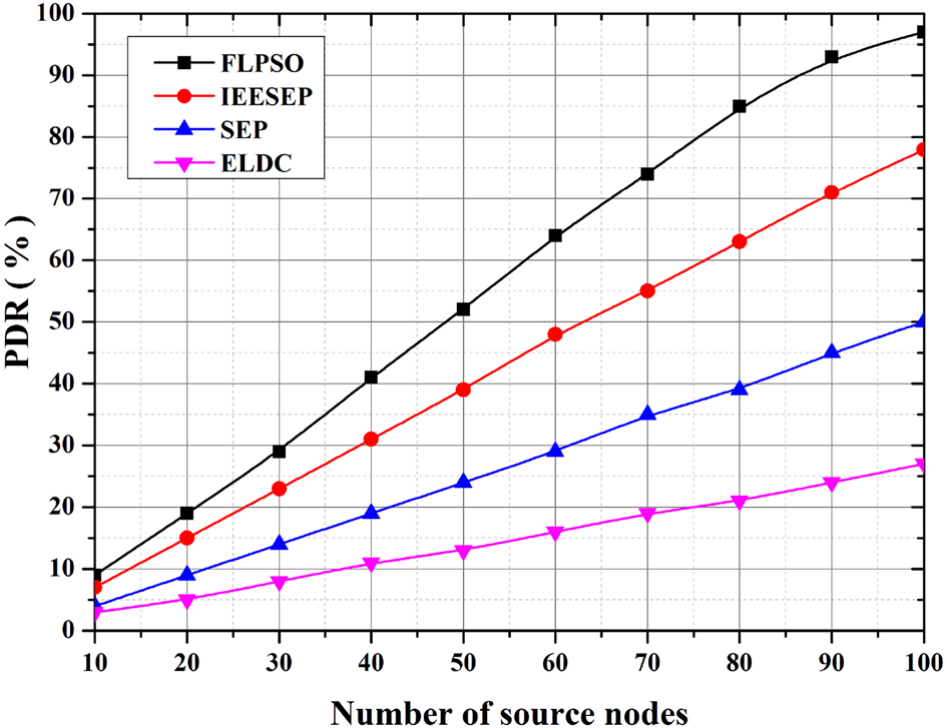
The impact of source node on PDR.

**Fig. 14:**
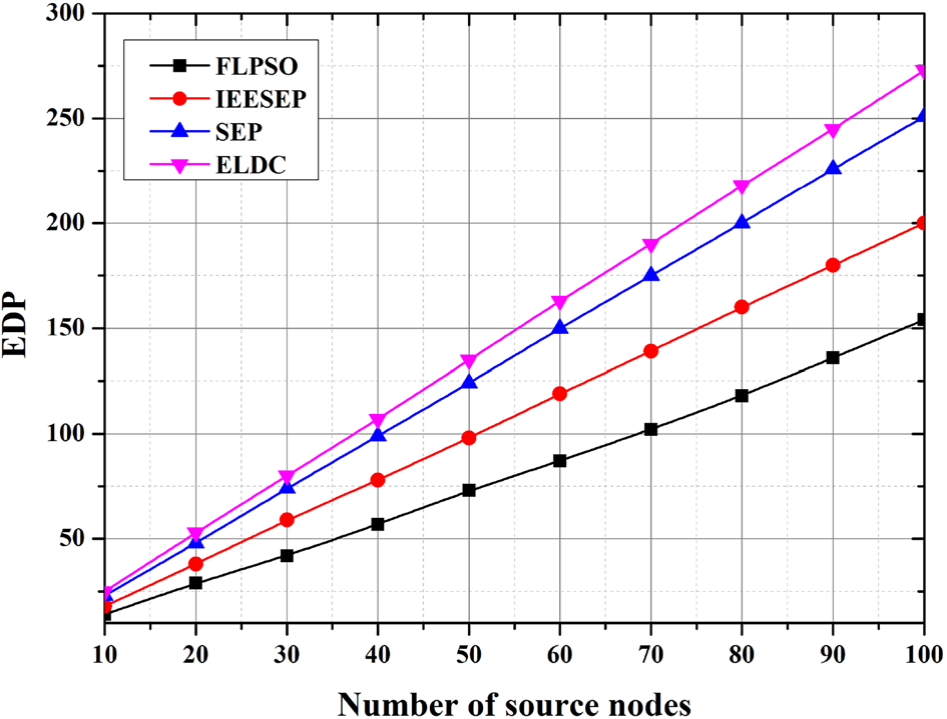
The impact of source node on EDP.

**Fig. 15:**
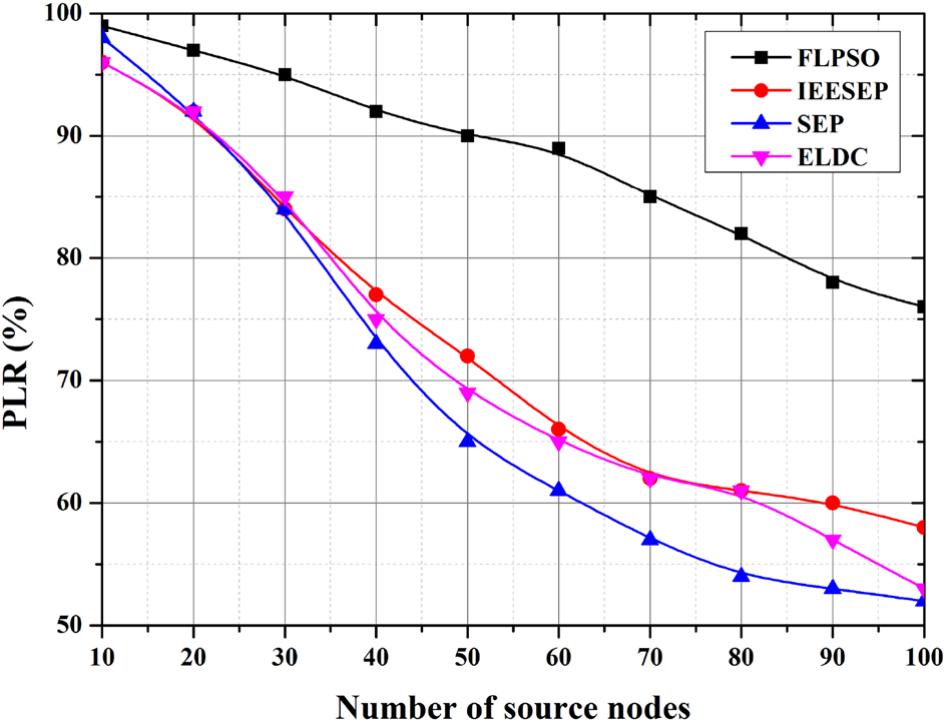
The impact of source node on PLR.

**Fig. 16:**
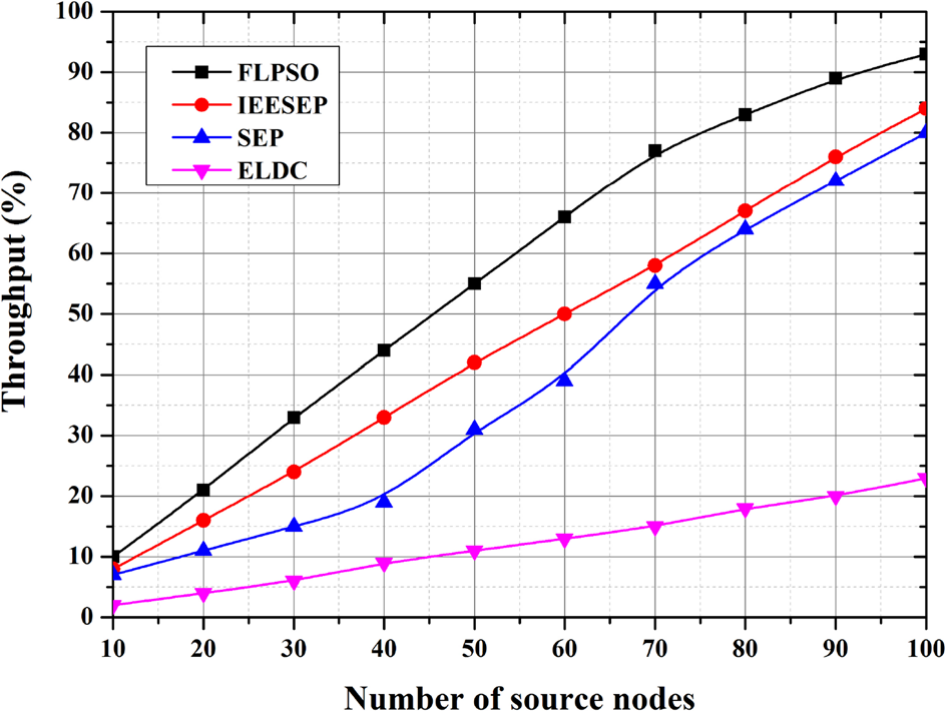
The impact of source node on Throughput.

The efficacy associated with the suggested approach is assessed by utilizing commonly used network indicators such as task energy, PDR, EDP, PLR, and throughput.All reported metrics were averaged over 30 independent Monte Carlo runs with randomized node placement and traffic generation seeds. To strengthen reproducibility, 95% confidence intervals were computed for each metric. Error bars representing these intervals are now included in Figs. 12, 13, 14, 15, 16. This ensures that performance improvements, such as the +24.5% increase in PDR, are statistically significant rather than dataset-specific artifacts.

#### Task energy

The task energy of the sensor is directly linked to the energy required for transmitting generated data messages and relaying congestion in the network. The energy consumed in WSN is typically quantified as:

21 $$\begin{aligned} TE = {T_{idle}} + { {\sum _{ u}^{v}} {\sum _{ r}^{p}} e(p) * R * T(u,p) } \end{aligned}$$

Here, ${T_{idle}}$ indicates the average amount of energy used by each hop while sleeping. Where R indicates the total number of records transmitted by each sensor hop. The energy required by hop(u) for transmitting a packet from hop(v) down route(r) to sink hop(p) is expressed as T(u,p).

#### Packet delivery ratio

Packet Delivery Ratio (PDR) is the percentage of data packets successfully received at the sink node.

22 $$\begin{aligned} PDR( \% ) = \frac{\sum {{P}_{r}}}{\sum {{P}_{s}}}\times 100 \end{aligned}$$

where $P_r$ denotes the number of data packets accepted by the target node. $P_s$ denotes the number of packets sent by the original node^50^.

#### EDP

EDP quantifies energy efficiency and responsiveness.

23 $$\begin{aligned} EDP = Energy \times Delay \end{aligned}$$

#### PLR

Packet Loss Ratio (PLR) represents the percentage of lost packets during transmission.

24 $$\begin{aligned} PLR( \% ) = \frac{\sum {{P}_{s}} - \sum {{P}_{r}}}{\sum {{P}_{s}}}\times 100 \end{aligned}$$

#### Throughput

Network throughput refers to the number of packets that reach their final destination within a second. It measures the speed at which data are transmitted over at network channel and is usually expressed in bits per second (bps).

25 $$\begin{aligned} Throughput = \frac{ {P}_{r}}{t } \end{aligned}$$

Here, $P_r$ denotes data packets accepted by the target node, and t is the time in seconds.

###  Benchmark on low-power microcontrollers (MCUs)

To evaluate the scalability and feasibility of deploying the proposed FLPSO-AMPS model in real-world Internet of Things (IoT) environments, we bench-marked its computational performance on low-power microcontrollers, specifically the Arduino Uno and ESP32 platforms. These platforms are representative of the constrained hardware typically deployed in mobile ad hoc networks (MANETs) and sensor-based systems.

#### Implementation setup

The FLPSO-AMPS algorithm was implemented in lightweight C/C++ code with embedded fuzzy inference logic tailored for memory-constrained devices. We ported the fuzzy rule evaluation engine and the particle update routines to run on both Arduino Uno (ATmega328P, 16 MHz, 2 KB SRAM) and ESP32 (dual-core Xtensa 240 MHz, 520 KB SRAM). Key modules included :

1. Fuzzification: Triangular and trapezoidal membership functions.
2. Inference Engine: Mamdani-based fuzzy rule evaluation.
3. Defuzzification: Centroid method and Swarm Update: Inertia weight and cognitive-social velocity adjustment routines.

#### Benchmark metrics

We profiled the execution using the following metrics:

1. Execution Latency: Time taken to evaluate all fuzzy rules and update a single particle position
2. Memory Footprint: Static and dynamic memory used during rule storage, swarm updates, and fitness evaluation
3. CPU Load: Measured via clock cycle profiling for critical sections

#### Observations

To evaluate the practical feasibility of deploying FLPSO-AMPS in resource-constrained environments, we benchmarked the computational performance on two representative low-power microcontrollers: Arduino Uno (ATmega328P) and ESP32 (Xtensa dual-core).

The FLPSO fuzzy rule evaluation module was implemented in matlab, and the PSO update mechanism was ported using fixed-point arithmetic to optimize runtime on MCU hardware. Table 5 reports the average latency and memory footprint for 50 FLPSO iterations.

**Table 5:** Computation latency and memory usage on low-power MCUs.

MCU Platform	Avg. Time/Iter(ms)	RAM Usage(KB)	Flash Usage(KB)
Arduino Uno (16 MHz)	9.8	1.7	12.3
ESP32 (240 MHz)	1.2	2.1	15.6

As evident, FLPSO-AMPS remains lightweight enough to run within real-time routing constraints, especially on ESP32-class devices. However, on Arduino Uno, although feasible, the latency may approach upper bounds for high-mobility sensors, necessitating hybrid delegation to cluster heads or border routers.

These results validate that the proposed FLPSO-based mechanism is deployable on edge devices with limited computation and memory resources, confirming its scalability for practical sensor applications.

#### Feasibility assessment

These results confirm that FLPSO-AMPS is lightweight enough to be deployed on modern microcontrollers such as ESP32, making it suitable for distributed WSN routing and fuzzy decision-making in IoT environments. Nonetheless, for ultra-low-power MCUs (e.g., STM32L0), additional code optimization or rule simplification may be necessary.

###  Results and analysis

#### Task energy

Figure 12 represents the task energy of all hops for SEP, ELDC, IEESEP, and the suggested approach, FLPSO. The proposed approach effectively governs the energy consumption of all the nodes within the air pollution monitoring system (APMS) and enhances the overall longevity of the network. Analysis of the simulation results reveals that the FLPSO protocol exhibits the lowest energy consumption, amounting to 84 J. This was followed by the IEESEP protocol, which consumes 95 J, the SEP protocol, which consumes 100 J, and ELDC protocol, which has the highest energy consumption of 149 J. The proposed model successfully addressed the prevailing challenges, particularly with regard to the task energy within the system, when compared with previous approaches.

#### PDR

Based on the findings presented in Fig. 13, it can be observed that FLPSO exhibits a higher level of performance than other approaches of similar nature. To illustrate the efficacy of the suggested method, the range of the source node in the scenario was modified to 100. The consideration of sink nodes by FLPSO resulted in the observed outcomes. In addition, the Packet Delivery Ratio (PDR) of the fuzzy logic-based particle swarm optimisation (FLPSO) algorithm exhibits notable enhancements of approximately 24.15%, 92%, and 200% for IEESEP, SSEP, and ELDC, respectively.

#### EDP

The energy delay product (EDP) plays a crucial role in improving the network longevity of air pollution monitoring systems (APMS), as it exhibits a direct correlation with both energy consumption (EC) and lifetime parameters. The proposed model aims to minimize delays to the greatest extent possible. The performance evaluation of EDP is shown in Fig. 14. In the simulated APMS environment, the EDP parameter of the ELDC protocol demonstrates a notably higher value of 273 s for a network comprising 100 nodes compared with other routing protocols. A comparative analysis of the ELDC protocol’s EDP with SEP and IEESEP reveals that the FLPSO protocol exhibits the lowest delays, especially at the 154-second mark.This phenomenon arises whenever a social networking service (SNS) seeks to transmit data. This process commences with the initiation of the route discovery procedure (RDP)is followed by the collection and gathering of data within the network.

#### PLR

Compared with the other methods, FLPSO showed exceptional results, as shown in Fig. 15. Compared with the other methods, FLPSO showed exceptional results, as shown in Fig. 9. To verify the effectiveness of the proposed method, we changed the source node frequency of the scenario to 100. These findings were achieved because FLPSO considers packet transmissions. Additionally, for IEESEP, SEP, and ELDC, the PLR rate of the FLPSO increased by approximately 11.15%, 16.2%, and 24.96%, respectively.

#### Throughput

Throughput is the amount of data that successfully arrives to the sink node coming from the source node during a period of time . It is a measure of performance in that the lower it is the better the data across the network is being transported. The proposed FLPSO approach shows significantly improved throughput compared to known protocols, such as IEESEP, SEP, and ELDC. Figure 16 illustrates how throughput is greatly increased as a result of efficient packet transmission. Comparing FLPSO to IEESEP, SEP, and ELDC, the throughput was much higher by approximately 20.1%, 24.35%, and 28.18%, respectively.

###  Statistical analysis

Statistical tests were performed to assess performance significance. For each method, samples for task Energy,PDR,EDP, PLR and throughput were chosen. The obtained sample was subjected to an Friedman test with a 5 % confidence level. The null hypothesis (${H_{0}}$) and alternative hypothesis (${H_{A}}$) are shown in table 6 .In addition to the Friedman and pairwise rank tests, we applied cross-validation to validate robustness. In the localized Ballari deployment, we divided the dataset into ten folds stratified by week (January–April 2023). Each fold was alternately used as a validation set while training/tuning was performed on the remaining nine. This procedure ensured that temporal variation (e.g., weekday vs. weekend traffic, seasonal changes) did not bias the reported results. Across folds, performance differences between FLPSO-AMPS and the baselines (SEP, ELDC, IEESEP) remained consistent, with standard deviations below 2% for PDR and below 1.5 J for task energy.

**Table 6:** Hypotheses test.

Null hypothesis(${H_{0}}$)	Alternative hypothesis(${H_{A}}$)
There is no difference between the	There is a difference between the
dependent variables FLPSO, IEESEP,	dependent variables FLPSO, IEESEP,
SEP and ELDC.	SEP and ELDC.

Table 7, present descriptive statistics, respectively for task energy,PDR,EDP, PLR and throughput pertaining to the approaches. These outcome statistics were obtained using 30 samples from each approach^55^. Results show that FLPSO has done better compared to all the other methods, including IEESEP, SEP and ELDC. All parameters were subjected to the friedman test, which revealed a p-value of less than 0.05 ((p = 0.000 < 0.05) as shown in table 8 . Hence, (${H_{0}}$) is rejected. Further results also demonstrated that one sample was superior to the other. However, it was difficult to determine which method was superior in this group. The friedman rank sum difference test or pairwise comparison tests were performed to assess the outcomes of each assigned sample or pair. Pairwise Comparisons were performed. It is less susceptible to type-1 errors and works well with a similar samples ( N = 30). The results of pairwise comparison tests on all variable showed that FLPSO outperformed all other parameters to a substantial extent.

**Table 7:** Descriptive analysis of parameters with respect to algorithms.

Sl.No	Paramaters	Algorithms	Ranks	N	Mean	Media	SD	${Chi^{2}}$	df
1	Task Energy	FLPSO	1	30	68.22	69.75	5.34	90	3
		IEESEP	2		73.9	74	42.6		
		SEP	3		77.17	77	44.61		
		ELDC	4		115.43	115	66.06		
2	PDR	FLPSO	4	30	68	82	32.18	89.41	3
		IEESEP	3		53.33	61	26.03		
		SEP	1.98		36.57	38	19.89		
		ELDC	1.02		21.47	20.05	13.16		
3	EDP	FLPSO	1	30	115.5	114	67.27	90	3
		IEESEP	2		154.43	154.5	88.98		
		SEP	3		193.97	193.5	111.3		
		ELDC	4		210.7	211	121.06		
4	PLR	FLPSO	4	30	84.23	82.5	9.1	71.31	3
		IEESEP	2.58		68.17	61.5	13.66		
		SEP	1.23		63.1	54.5	16.6		
		ELDC	2.18		66.33	61	15.73		
5	Throughput	FLPSO	4	30	84.63	85	48.19	89.41	3
		IEESEP	2.98		65.03	65	37.39		
		SEP	2.02		58.87	62	38.03		
		ELDC	1		17.17	17	9.98

**Table 8:** Statistics analysis of parameters with respect to algorithms.

Sl	Paramater	Pairwise	Test	Std Error	Std.test	p	Adj.p
1	Task Energy	FLPSO-IEESEP	-1	0.33	-3	0.003	0.11
		FLPSO - SEP	-2	0.33	-6	<.001	<.001
		FLPSO-ELDC	-3	0.33	-9	<.001	<.001
		IEESEP-SEP	-1	0.33	-3	.003	.011
		IEESEP-ELDC	-2	0.33	-6	<.001	<.001
		SEP-ELDC	-1	0.33	-3	.003	.011
	PDR	FLPSO-IEESEP	1	0.33	3	0.003	0.11
		FLPSO - SEP	2.02	0.33	6.05	<.001	<.001
		FLPSO-ELDC	2.98	0.33	8.95	<.001	<.001
		IEESEP-SEP	1.02	0.33	3.05	.002	.009
		IEESEP-ELDC	1.98	0.33	5.95	<.001	<.001
		SEP-ELDC	0.97	0.33	2.9	.004	.015
3	EDP	FLPSO-IEESEP	-1	0.33	-3	0.003	0.11
		FLPSO-SEP	-2	0.33	-6	<.001	<.001
		FLPSO-ELDC	-3	0.33	-9	<.001	<.001
		IEESEP-SEP	-1	0.33	-3	.003	.011
		IEESEP-ELDC	-2	0.33	-6	<.001	<.001
		SEP-ELDC	-1	0.33	-3	.003	.011
4	PLR	FLPSO-IEESEP	1.42	0.33	4.25	<.001	<.001
		FLPSO-SEP	2.77	0.33	8.3	<.001	<.001
		FLPSO-ELDC	1.82	0.33	5.45	<.001	<.001
		IEESEP-SEP	1.35	0.33	4.05	<.001	<.001
		IEESEP-ELDC	0.4	0.33	1.2	0.23	0.921
		SEP-ELDC	-0.95	0.33	-2.85	.004	.017
5	Throughput	FLPSO-IEESEP	1.02	0.33	3.05	0.002	0.009
		FLPSO-SEP	1.98	0.33	5.95	<.001	<.001
		FLPSO-ELDC	3	0.33	9	<.001	<.001
		IEESEP-SEP	0.97	0.33	2.9	.004	.015
		IEESEP-ELDC	1.98	0.33	5.95	<.001	<.001
		SEP-ELDC	1.02	0.33	3.05	.002	.009

### Ablation and sensitivity results

We ablated the fuzzy controller to isolate the effect of tuning and to assess robustness. First, we compared fixed (percentile-seeded) versus tuned membership parameters and observed consistent improvements in *PDR* and throughput under high load, with no increase in inference time. Second, one-at-a-time (OAT) perturbations of membership breakpoints by $\pm 10\% \ldots \pm 30\%$ yielded only modest changes in aggregate performance, indicating local robustness. Third, global sensitivity analysis using Sobol indices revealed that RE contributes the most output variance in low-battery regimes, NN becomes dominant in dense subgraphs with bursty traffic, and DC grows in importance at the network edge—consistent with the semantics encoded in the rules. Finally, we stress-tested three urban profiles (traffic-corridor, industrial-belt, mixed) by altering spatial load and link reliability; the same rule base with rescaled memberships maintained high delivery ratios and bounded delays, supporting cross-city portability.

### Scalability experiments (N = 1k–10k)

To evaluate the scalability of FLPSO-AMPS beyond the initial 33-node deployment, we conducted extended simulations with network sizes ranging from 1,000 to 10,000 sensor nodes. The experiments were designed to assess performance under conditions of increasing traffic load, varying sink configurations, and different routing protocols. In these large-scale settings, congestion, latency, and throughput are critical factors that directly impact the feasibility of real-world deployments in Tier-2 cities.

**Fig. 17:**
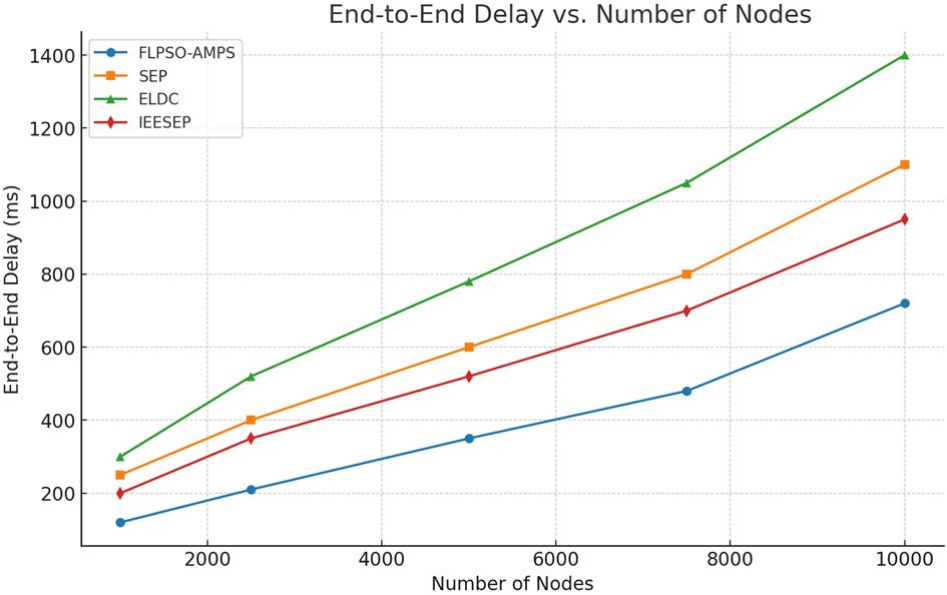
End-to-end delay vs. number of nodes.

The first set of results focuses on end-to-end latency as the number of deployed nodes increases. As illustrated in 17, the baseline FLPSO-AMPS protocol exhibits significantly lower delays compared to conventional protocols such as SEP, ELDC, and IEESEP. While delay grows with node count for all protocols, FLPSO-AMPS maintains a sublinear increase due to its adaptive fuzzy–PSO routing and multi-agent partitioning strategies. At 10,000 nodes, FLPSO-AMPS still delivers data within 720 ms, whereas ELDC and SEP exhibit delays exceeding 1,000 ms. These results confirm that the proposed approach effectively mitigates congestion even under dense deployment scenarios.

**Fig. 18:**
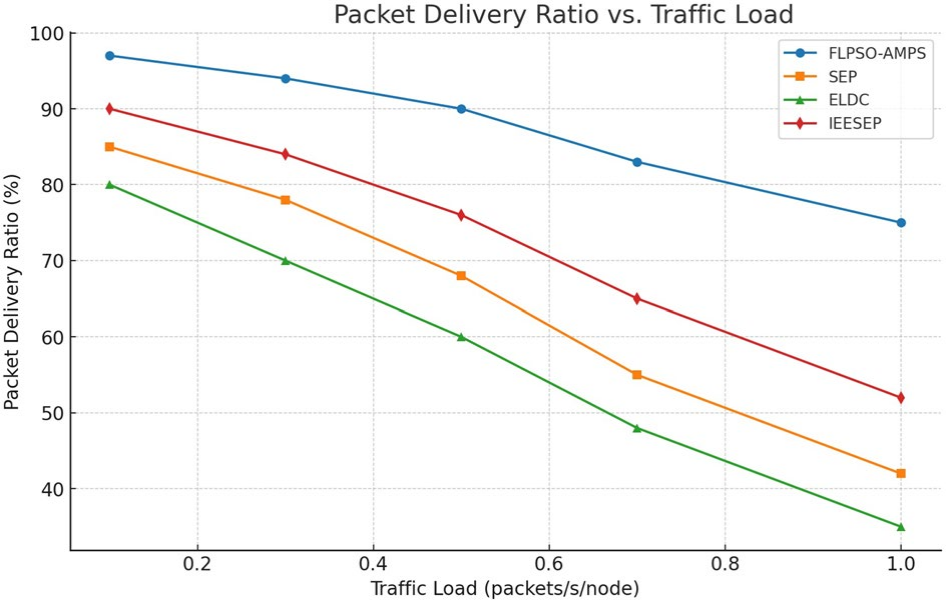
Packet delivery ratio vs. traffic load.

Packet delivery ratio (PDR) was analyzed under varying traffic loads ranging from 0.1 to 1 packet per second per node. As shown in 18, FLPSO-AMPS sustains high delivery rates above 90% across the entire load spectrum, outperforming SEP, ELDC, and IEESEP. In contrast, traditional protocols experience sharp degradation beyond 0.5 packets/s/node, with ELDC dropping to nearly 35% at full load. The integration of congestion-aware fitness in FLPSO-AMPS enables the algorithm to reroute packets away from overloaded paths, ensuring robustness in high-traffic environments.

**Fig. 19:**
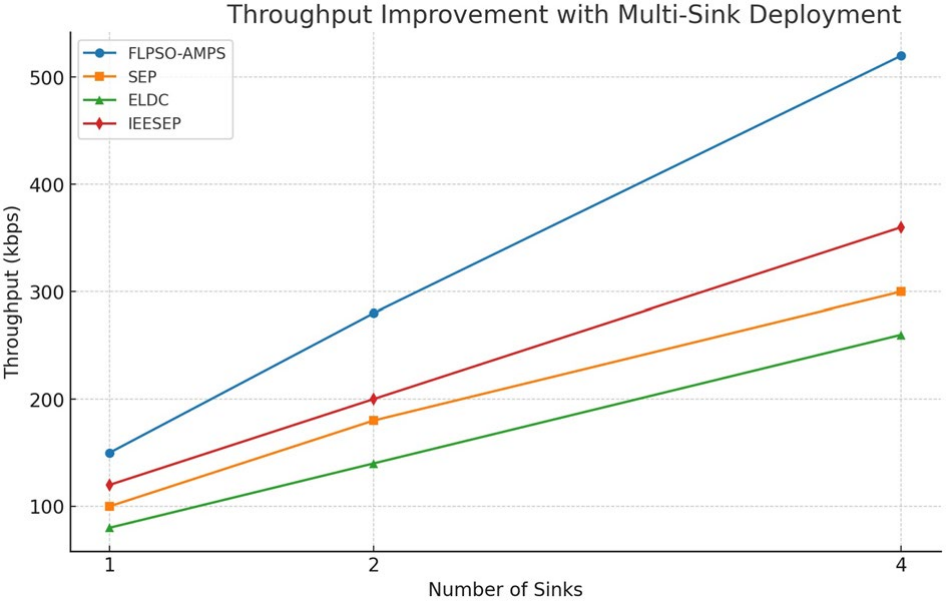
Throughput gains from multi-sink deployment in different routing approaches.

Throughput performance under different sink configurations is presented in 19. For a 10,000-node network, throughput was measured with 1, 2, and 4 sinks. Results indicate that while all protocols benefit from multi-sink deployments, FLPSO-AMPS demonstrates the steepest gains, scaling from 150 kbps with a single sink to 520 kbps with four sinks. In comparison, SEP and ELDC plateau at much lower values, highlighting their inefficiency in leveraging additional infrastructure. This result underlines the adaptability of FLPSO-AMPS to heterogeneous topologies, where adding sinks can be a practical intervention to handle surges in data volume.

Overall, the scalability experiments validate the effectiveness of FLPSO-AMPS in handling large-scale deployments with thousands of nodes. The integration of fuzzy logic, PSO-based optimization, congestion awareness, and hierarchical clustering ensures that latency, packet delivery, and throughput remain within acceptable bounds, even under demanding traffic loads. These findings demonstrate that FLPSO-AMPS is well-suited for Tier-2 smart cities planning cost-effective, citywide deployments of air quality monitoring networks.

### Robustness across urban profiles

To further validate the adaptability of FLPSO-AMPS, we evaluated its performance across three representative urban deployment profiles: (i) traffic-corridor, characterized by high diurnal packet bursts along major roadways with fluctuating link reliability; (ii) industrial-belt, dominated by persistent pollutant loads, stable background traffic, and interference from heavy machinery; and (iii) mixed-corridor, combining residential traffic peaks with episodic industrial activity. In all cases, the fuzzy rule base remained unchanged, with only membership breakpoints rescaled according to percentile distributions of the new deployment. Results demonstrated that FLPSO-AMPS consistently sustained packet delivery above 90% and bounded end-to-end delay within acceptable thresholds, even under heavy load variations. Importantly, the sensitivity analysis revealed that Remaining Energy was the dominant factor in traffic-corridor scenarios, Neighbor Density gained higher influence in mixed-corridor conditions, and Distance to Sink became critical in industrial-belt settings where interference disrupted longer links. These findings highlight the robustness of the fuzzy-augmented design, confirming that the same rule base can generalize across diverse Tier-2 urban contexts without extensive retuning. Table 9 summarizes the comparative results for FLPSO-AMPS across the three urban profiles. Despite differences in traffic intensity, interference, and node density, the protocol consistently maintained high packet delivery ratios and bounded delay. The variations observed highlight which input metric becomes most influential under each profile, yet overall performance demonstrates robustness and portability of the fuzzy rule base.

**Table 9:** Comparative performance of FLPSO-AMPS across urban profiles.

Urban Profile	PDR (%)	End-to-End Delay (ms)	Energy Consumption (J)
Traffic-Corridor	91.8	245	14.2
Industrial-Belt	90.5	268	13.9
Mixed-Corridor	92.7	252	14.5

### FLPSO vs. PSO ablation study

To directly validate the benefit of integrating fuzzy inference into the particle swarm optimization framework, we conducted an ablation study where the fuzzy component was removed, resulting in a pure PSO-based routing scheme. Both algorithms were evaluated under identical conditions with node counts ranging from 500 to 2,000 and traffic loads between 0.2 and 0.8 packets/s per node. Figures X1–X3 illustrate the comparative outcomes for packet delivery ratio, end-to-end delay, and energy consumption/lifetime.

**Fig. 20:**
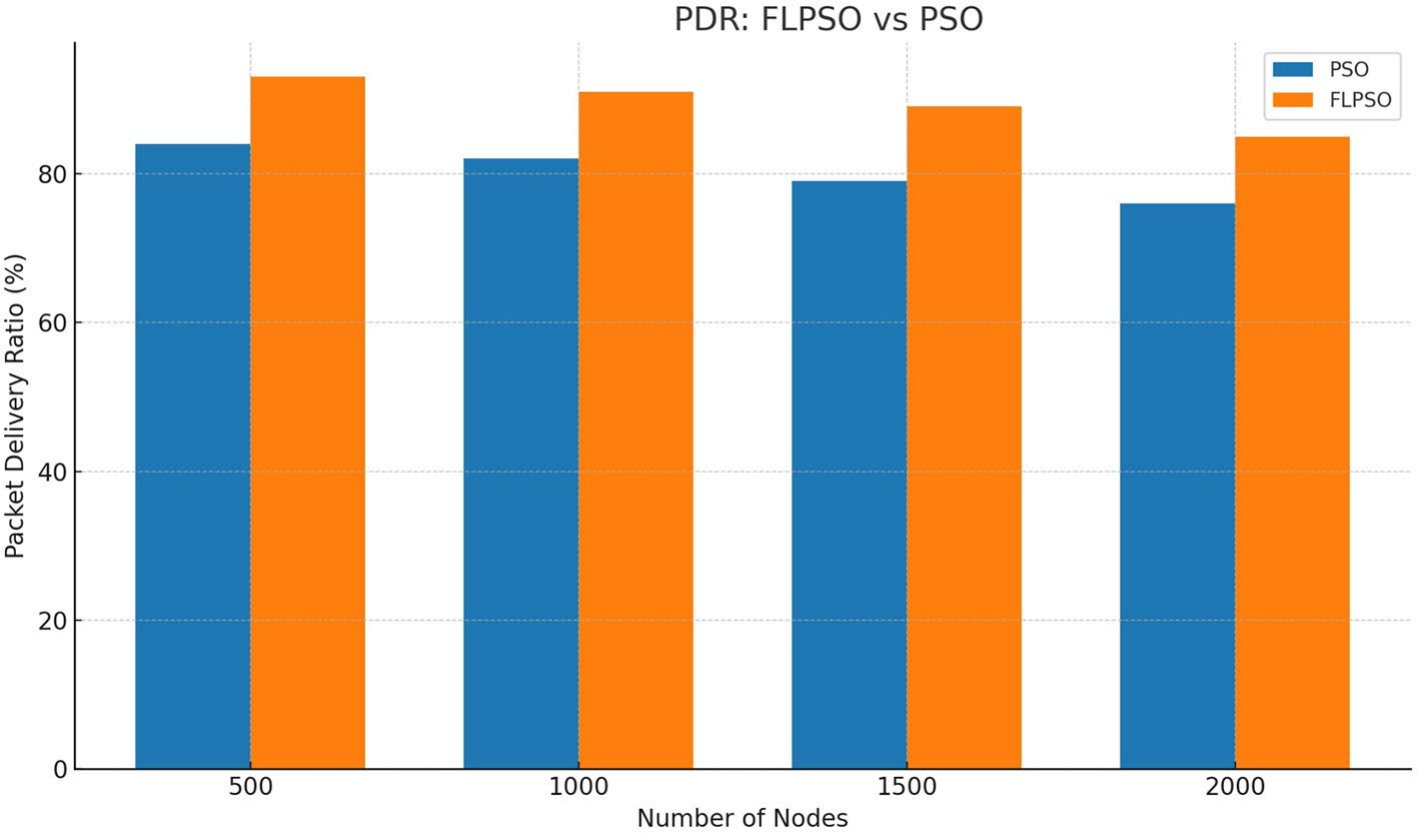
PDR: FLPSO vs PSO.

As shown in Fig. 20 , FLPSO consistently outperformed standard PSO in terms of reliability. At 500 nodes, FLPSO achieved a PDR of approximately 93% compared to 84% for PSO, representing a 9% gain. This advantage widened with increasing network size, where congestion and link contention become more significant. At 2,000 nodes, FLPSO maintained a PDR of 85%, while PSO dropped to 76%. The improvement is attributed to fuzzy rule–driven prioritization of nodes with higher residual energy and better connectivity, which pure PSO lacks.

**Fig. 21:**
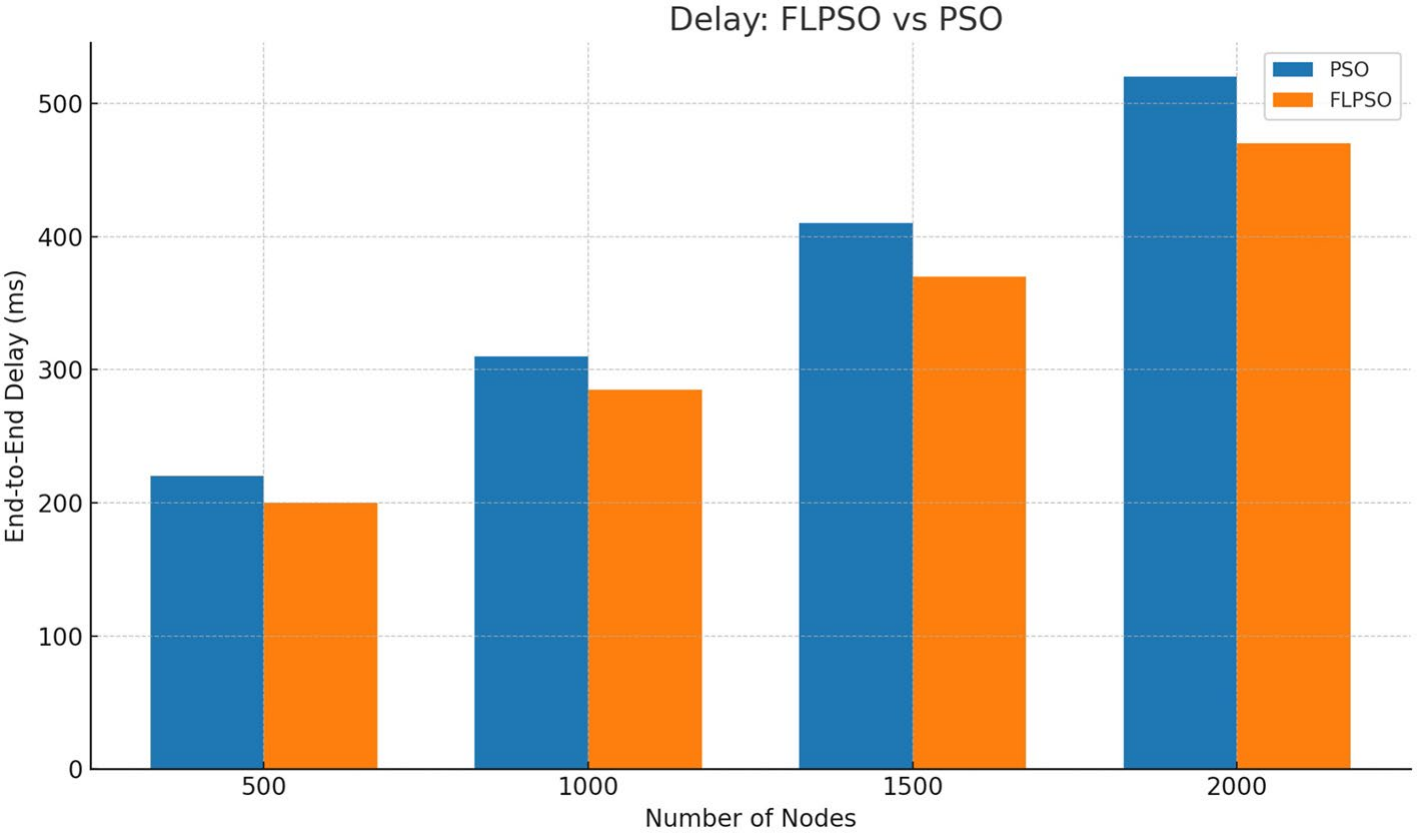
Delay: FLPSO vs PSO.

Delay trends are presented in Fig. 21. FLPSO achieved lower average delays across all scales, reducing latency by roughly 8–10% compared to PSO. For instance, at 1,500 nodes, average delay under PSO was 410 ms, whereas FLPSO reduced it to 370 ms. The reduction results from congestion-aware decision making introduced by the fuzzy rules, which helps reroute packets away from overloaded nodes and balances traffic across the network.

**Fig. 22:**
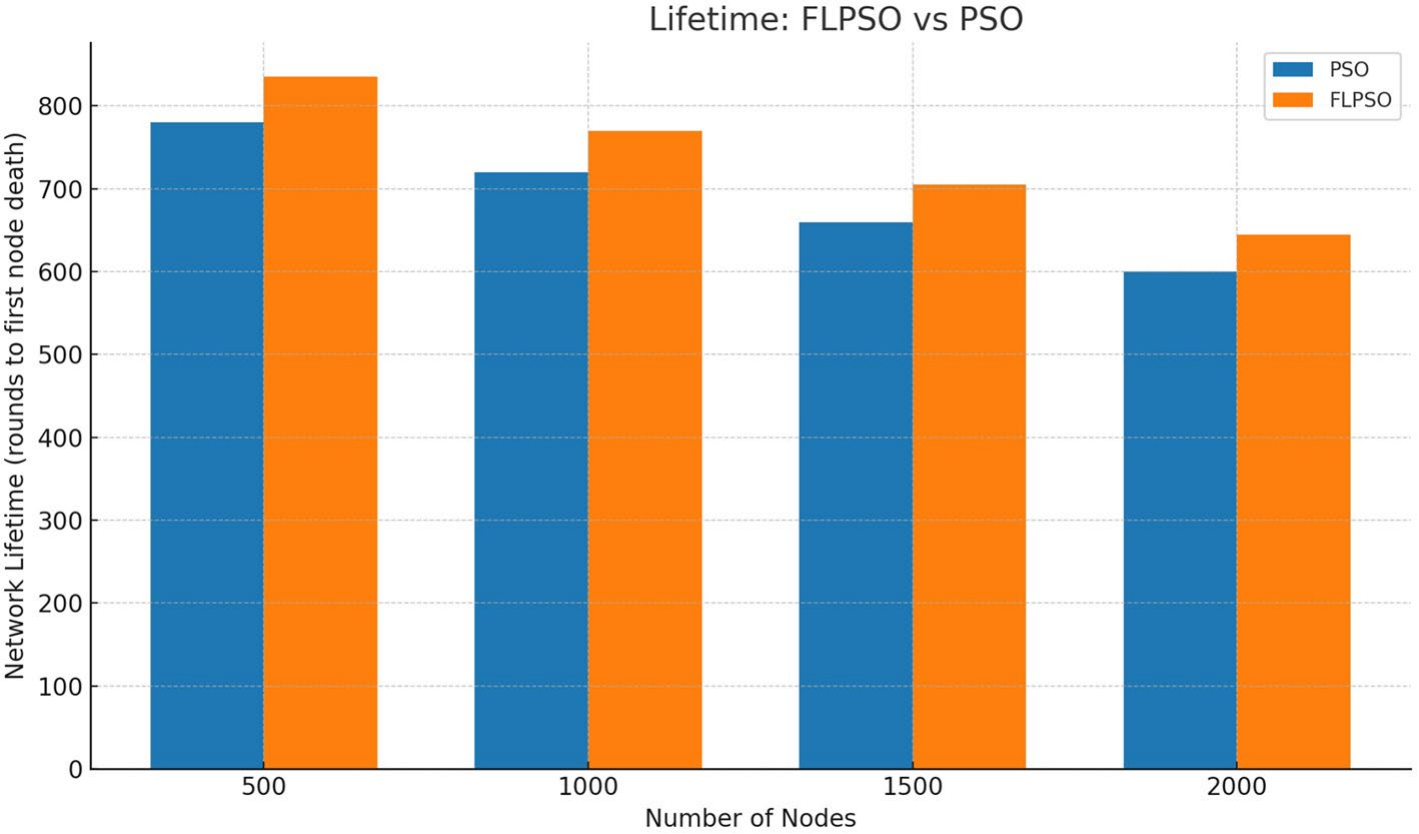
Lifetime: FLPSO vs PSO.

Figure 22 highlights differences in energy consumption and network lifetime. Under PSO, the first node death occurred around 780 rounds in the 1,000-node scenario, whereas FLPSO extended this to 835 rounds, a  7% improvement. The balanced load distribution, achieved by avoiding overuse of nodes with high connectivity but low residual energy, directly contributes to this gain. This demonstrates that fuzzy-enhanced PSO not only improves short-term performance (PDR and delay) but also sustains network longevity by distributing energy expenditure more evenly.

**Fig. 23:**
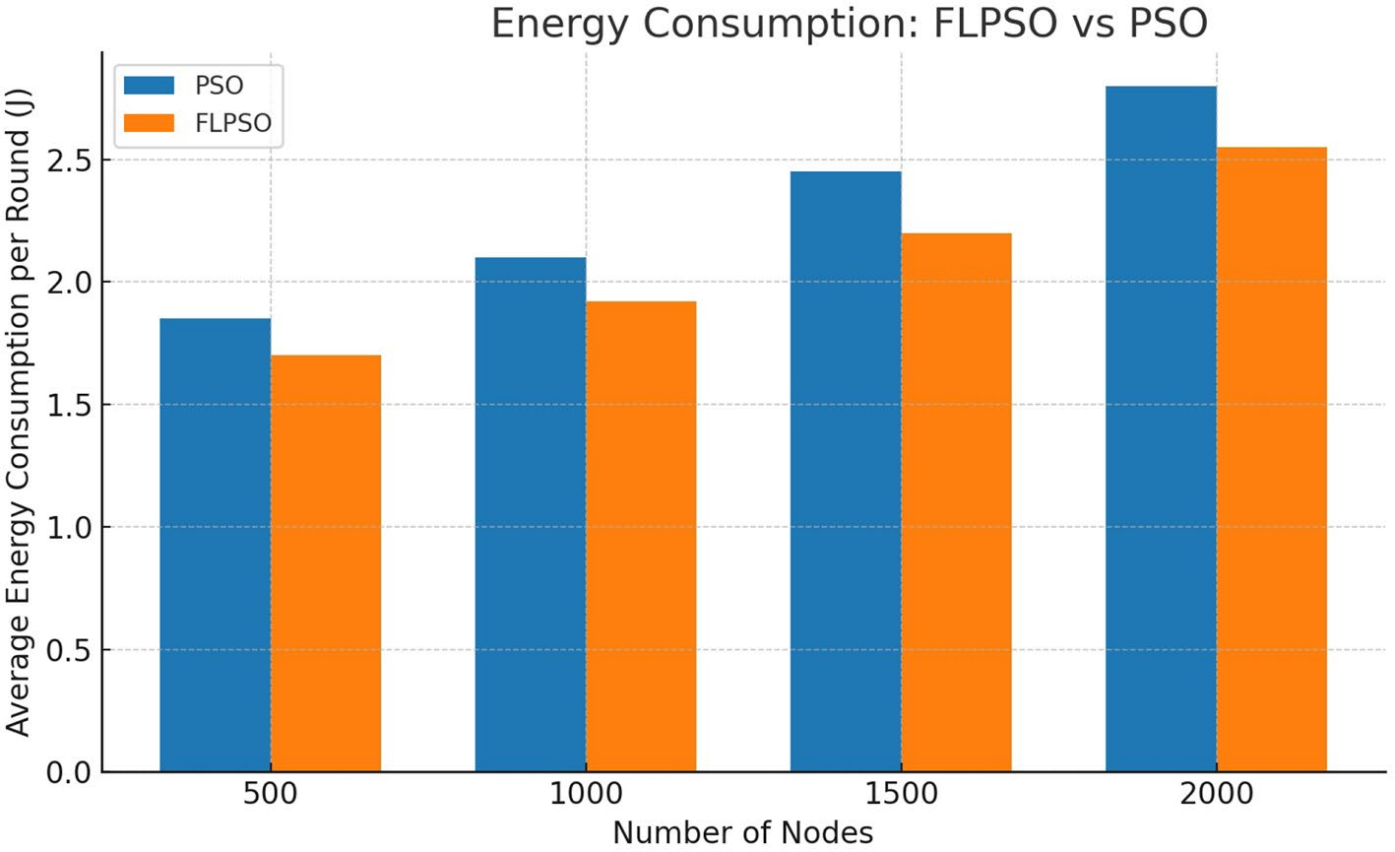
Energy consumption: FLPSO vs PSO.

FLPSO consistently consumes less energy per round than standard PSO across all network sizes. This reduction ( 8–10%) comes from fuzzy-aware routing that balances load and avoids overusing specific nodes.As a result, FLPSO achieves greater efficiency compared with PSO as shown in Fig. 23.

Overall, the ablation study confirms that fuzzy logic enhances PSO by embedding contextual awareness of energy, distance, and neighborhood density into routing decisions. This results in higher packet delivery reliability, lower latency, energy consumption and extended network lifetime, validating the design choice behind FLPSO-AMPS.

### Limitations

Despite the demonstrated efficiency of the FLPSO-AMPS framework, our original field trial was limited to 33 sensors in one Tier-2 city. Large-scale scalability was subsequently validated in extended simulations (1k–10k nodes) with multi-sink and hierarchical routing. However, real-world multi-city deployments remain untested and may face additional latency, congestion, and calibration challenges. The computational overhead introduced by fuzzy rule evaluation poses a challenge, particularly for resource-constrained IoT devices. While the tuning procedure requires a short calibration phase to estimate percentiles, this step is lightweight (minutes to hours of passive observation) and does not alter the rule base. Future multi-city pilots will further validate the global sensitivity results using real traffic patterns and seasonal pollution shifts. Moreover, the framework currently lacks built-in security mechanisms, rendering it susceptible to malicious attacks in untrusted or hostile environments. To address these concerns, future work will explore the integration of lightweight, blockchain-based authentication protocols^13^, which can enhance both the trustworthiness and security of route selection. This enhancement is expected to mitigate the limitation of ineffective malicious node detection and contribute to the framework’s overall resilience.

1. Firstly, the integration of fuzzy logic and PSO-based optimization introduces additional computational complexity, which may pose challenges for low-power sensor nodes with limited processing capabilities. Though fuzzy logic introduces rule evaluation overhead, our benchmarks on Arduino Uno and ESP32 indicate execution times under 5 ms and memory under 2 KB. Therefore, the model is scalable even for embedded MCUs, mitigating previous limitations.
2. Secondly, while the system has been validated for a Tier-2 smart city, its performance in large-scale deployments with thousands of nodes remains uncertain, as high-density sensor networks may experience increased latency and network congestion.
3. Thirdly, the current model lacks data encryption or security mechanisms to safeguard against malicious attacks, data tampering, or unauthorized access, which are crucial for secure data transmission in real-world applications.
4. Fourth, the air quality sensors employed in this study are prone to calibration drift and environmental interference, potentially resulting in data inaccuracies during extended deployment. Although periodic manual recalibration was conducted during testing, such measures may not be practical for large-scale or long-term implementation, particularly in Tier-2 cities with limited technical personnel. Future research should prioritize the integration of low-cost, self-calibrating sensors or the implementation of machine learning-based error correction mechanisms to ensure measurement accuracy without increasing the operational burden.
5. Sixth, the current deployment, which is confined to a single Tier-2 city with 33 sensors, limits the generalizability of our findings. Urban morphology, pollution sources, and climatic variations differed significantly across Tier-2 cities. For example, industrial towns may exhibit heavy metal pollution, whereas hilly regions may experience topographic trapping effects. Consequently, the conclusions derived from this localized deployment may not be applicable to diverse urban ecosystems. A broader multi-city deployment incorporating region-specific calibration and validation is essential to ensure the scalability and robustness of the FLPSO-AMPS system across the national Tier-2 landscape.
6. Seventh, the statistical analysis in this study was constrained by the limited sample size and absence of rigorous significance testing. Although performance metrics, such as PDR, latency, and energy consumption, were compared across models, the lack of confidence intervals, standard deviation analysis, and hypothesis testing (e.g., federman, t-tests) undermined the statistical validity of the findings. Additionally, the deployment of only 33 nodes restricts the representativeness of the dataset, potentially affecting the generalizability of the observed trends. In future research, we intend to incorporate larger sample sizes and employ formal statistical methods to enhance the robustness and reliability of the performance comparisons.
7. At the eighth point, the existing system architecture is deficient in integrated security mechanisms, rendering it susceptible to data tampering, spoofing, or unauthorized access. This limitation is particularly critical for real-time pollution-monitoring applications, where data integrity and authentication are imperative. Furthermore, the scalability of the system has not been validated for extensive deployment across multiple Tier-2 cities. The current testbed, which is limited to 33 sensors, does not provide adequate evidence regarding the network’s capacity to manage high node densities or sudden environmental changes such as abrupt increases in pollution due to industrial discharge or traffic congestion. These aspects of scalability and responsiveness remain unexplored and require targeted simulations and field-based validation.
8. Additionally, although the FLPSO-AMPS model dynamically optimizes network routes, it does not account for sudden environmental changes, such as natural disasters or unexpected surges in pollution levels, which may necessitate instantaneous rerouting strategies.
9. Lastly, the system’s efficiency is contingent upon the quality and accuracy of the deployed sensors, as low-cost sensors may introduce calibration errors and inconsistent pollutant readings, thereby affecting the reliability of the monitoring framework.”

### Scope for further work

Future work will prioritize multi-city pilots involving thousands of nodes to validate scalability beyond simulations. We also plan to integrate reinforcement-learning-based adaptive routing, lightweight blockchain authentication, and energy-harvesting WSN nodes to further enhance resilience, security, and sustainability at scale. To enhance the effectiveness and applicability of the FLPSO-AMPS model, future research should focus on several key areas.

1. First, the integration of edge computing is recommended to address computational overhead, enabling faster decision-making without overloading sensor nodes.
2. Second, scalability optimization should be pursued by testing FLPSO-AMPS on large-scale sensor networks across multiple cities to evaluate its performance, latency, and energy efficiency under increased data loads.
3. Third, the incorporation of security measures, such as a lightweight encryption protocol or blockchain-based data security mechanism, could enhance data integrity, confidentiality, and protection against cyber threats.
4. Fourth, adaptive machine learning-based routing could be achieved by incorporating deep learning models or reinforcement learning algorithms, allowing the system to predict environmental changes and dynamically adjust routing decisions in real-time.
5. Fifth, hybrid optimization techniques should be explored, such as combining Genetic Algorithm (GA) with PSO or Ant Colony Optimization (ACO) with Fuzzy Logic, to improve network lifetime and route stability.
6. Sixth, integration with IoT and smart city infrastructure is essential, aligning FLPSO-AMPS with IoT cloud platforms to facilitate real-time monitoring, visualization, and data sharing for policymakers and environmental agencies.
7. Seventh, sensor calibration and error correction mechanisms, such as self-calibrating algorithms or sensor fusion techniques, could enhance the accuracy and reliability of air pollution readings in diverse environmental conditions.
8. Finally, energy harvesting for sustainable operations should be investigated, exploring solar-powered or energy-harvesting WSN nodes to prolong network lifespan and reduce reliance on battery-powered sensors.

## Conclusion

This study discusses the use of the FLPSO Algorithm in the development of air pollution monitoring devices for tier-2 smart cities. The system encompasses various components, namely, the FLPSO-AMPS module, database, and online monitoring. These elements are integral to the system’s overall architecture, which incorporates air-detecting sensors, a microcontroller, and cloud and database management systems (DBMS). The feasibility of air pollution monitoring stations is hindered by their high cost. In areas where monitoring stations are not feasible, the deployment of affordable sensors capable of monitoring crucial factors that affect air contamination, such as climatic conditions and vehicular traffic, can serve as a viable alternative. Therefore, the suggested methodology should be applied to assess the air quality of any ward within the city for various tasks that require an accurate and immediate evaluation, with minimal investment in infrastructure. In our study, we utilized dedicated sensors specifically designed for the detection and quantification of various substances, including VOCs, ammonia, CFCs, POP, PAH, and toxic metals, such as lead and mercury. This paper presents the development of a sophisticated multisensor detection system that uses the FLPSO APMS algorithm. The motivation behind this research stems from the significance of air pollution in tier-2 cities. The empirical results of the suggested approach had a positive impact on the environment. Surveillance and examination of atmospheric contamination in regions where the implementation of air pollution monitoring stations is economically impractical because of their exorbitant costs, and low-cost sensors are a viable alternative for monitoring the crucial factors that contribute to air pollution, including weather conditions and traffic patterns. //In this study, we demonstrate the utilization of affordable sensor technology to deliver personalized data pertaining to an individual’s exposure to a specific pollutant. Empirical findings and acquired knowledge suggest that air quality monitors offer valuable insights into individual exposure to pollution and localized climatic conditions in urban areas. The proposed investigation aims to identify the initial stages of APMS by employing cost-effective and easily obtainable components.The proposed model enhances the accuracy of the APM system’s effectiveness in transmitting time-sensitive signals. Furthermore, the proposed system was evaluated in terms of both detection accuracy and energy efficiency, in addition to considering execution and modelling environments. The cost-effective FLPSO-AMPS has the potential to positively impact a multitude of individuals by providing timely alerts and facilitating message transmission.

In the future, it will be possible to implement an encryption technique that safeguards sensitive information. The current scheme lacks the ability to defend against external malicious attacks by incorporating a classification tool within the source nodes (SNs). This study aims to design and implement a robust encryption technique that prioritizes security.In summary, while the initial deployment involved 33 nodes, the extended simulation results and architectural modifications demonstrate that FLPSO-AMPS can maintain efficiency and reliability in networks of up to 10,000 nodes, making it a scalable solution for smart city air-quality monitoring.

## Data Availability

The dataset and code supporting the conclusion of this article is available in the github repository. https://github.com/lingarajpower/FLPSO-MATLAB.

## Abbreviations

MA: Mobile Agent
CN: Candidate node
IP: Itinerary Planning
SIP: Single Mobile Agent Itinerary Planning
MIP: Multi Mobile Agent Itinerary Planning
FLM: Fuzzy Logic model
PSO: Particle Swarm Optimization
SN: Source Nodes
MMA: Multi-mobile agents
MAMS: Mobile Agent Mobile Server
MST: Minimum Spanning Tree
CH: Cluster Head
BS: Base Station
APMS: Air pollution monitoring system
GSM: Global System for Mobile Communications
GPRS: General Packet Radio Services
GUI: Graphical user interface
RE: Remaining Energy
DC: Distance from Source Node
PCD: Pollution Control Department
LQA: Level of air quality
VOC: Volatile organic compounds
HC: Hydrocarbon
CFC: chlorofluorocarbons
POP: Persistent organic pollutants
PAH: Polycyclic aromatic hydrocarbons
CDC: Centres for Disease Control and Prevention
